# A Novel N-Terminal Domain May Dictate the Glucose Response of Mondo Proteins

**DOI:** 10.1371/journal.pone.0034803

**Published:** 2012-04-10

**Authors:** Lisa G. McFerrin, William R. Atchley

**Affiliations:** 1 Bioinformatics Research Center, North Carolina State University, Raleigh, North Carolina, United States of America; 2 Department of Genetics, North Carolina State University, Raleigh, North Carolina, United States of America; University of South Florida College of Medicine, United States of America

## Abstract

Glucose is a fundamental energy source for both prokaryotes and eukaryotes. The balance between glucose utilization and storage is integral for proper energy homeostasis, and defects are associated with several diseases, e.g. type II diabetes. In vertebrates, the transcription factor ChREBP is a major component in glucose metabolism, while its ortholog MondoA is involved in glucose uptake. Both MondoA and ChREBP contain five Mondo conserved regions (*MCRI-V*) that affect their cellular localization and transactivation ability. While phosphorylation has been shown to affect ChREBP function, the mechanisms controlling glucose response of both ChREBP and MondoA remain elusive. By incorporating sequence analysis techniques, structure predictions, and functional annotations, we synthesized data surrounding Mondo family proteins into a cohesive, accurate, and general model involving the *MCR*s and two additional domains that determine ChREBP and MondoA glucose response. Paramount, we identified a conserved motif within the transactivation region of Mondo family proteins and propose that this motif interacts with the phosphorylated form of glucose. In addition, we discovered a putative nuclear receptor box in non-vertebrate Mondo and vertebrate ChREBP sequences that reveals a potentially novel interaction with nuclear receptors. These interactions are likely involved in altering ChREBP and MondoA conformation to form an active complex and induce transcription of genes involved in glucose metabolism and lipogenesis.

## Introduction

Glucose is a carbohydrate in the form of a simple sugar that is an important source of energy for both eukaryotes and prokaryotes. However, glucose regulation is complex and not well understood. Extensive work has been devoted to the function of individual components within known metabolic pathways, e.g. [1]–[3], yet our understanding of their coordinated roles in response to different metabolic and cancerous conditions is just beginning to take shape. The discovery of additional regulatory factors such as ChREBP and MondoA broach this issue, but still need to be incorporated in current models of glucose sensing and regulation.

### SREBF1 and ChREBP promote glucose storage in mammals

In mammals, the liver is the primary organ that controls energy homeostasis by processing glucose for energy or storage. In fasting conditions, the liver produces glucose via *de novo* synthesis (gluconeogenesis) or decomposition of glycogen (glycogeneolysis). Glucose can then be converted to pyruvate through glycolysis and subsequently enter the citric acid (TCA) cycle within mitochondria to produce energy. In contrast, when excess carbohydrates are consumed, glucose can be stored according to two major pathways. Insulin induced enzymes trigger the glycogen synthase pathway to store glucose as glycogen. Alternatively, glucose can be converted to triglycerides through the *de novo* lipogenesis pathway for a more compact form of storage. Triglycerides within the liver can be further packaged into lipoproteins (i.e. VLDL, LDL, HDL) and transported into the blood stream and other tissues.

Initially, sterol regulatory elemenent binding transcription factor 1 (SREBF1) was identified as the major factor involved in glucose metabolism and insulin response [4]. However, knockout experiments revealed an additional factor was necessary for the full glucose-dependent transactivation of certain lipogenic genes, e.g. acetyl-CoA carboxylase (ACC) and fatty acid synthase (FAS) [5]–[7]. The discovery of a conserved carbohydrate response element (ChORE) consisting of two E-boxes separated by exactly 5 residues (CACGTGN_5_CACGTG) within the promoters of such genes facilitated the identification of this glucose responsive element [8]; ChORE binding protein ChREBP has subsequently been implicated in transactivation of several genes that regulate the *de novo* lipogenesis pathway, e.g. liver pyruvate kinase (L-PK), malic enzyme (ME), glucose phosphoisomerase (GPI), ACC, and FAS [9].

ChREBP protein, also named WBSCR14, MondoB and MLXIPL, has a paralog in vertebrates named MondoA or MLXIP. Interestingly, MondoA and ChREBP have overlapping yet distinct expression profiles, which underly their downstream effects and separate roles in regulating genes involved in glucose metabolism. MondoA can restrict glucose uptake and influences energy utilization, while ChREBP signals energy storage through *de novo* lipogenesis [10], [11]. Only a single Mondo gene has been identified in invertebrate animals [12], including *Drosophila melanogaster* (*dmondo/mio*) and *Caenorhabditis elegans* (*mml-1/T20B12.6*) [13]. We refer to this single ortholog in invertebrates as non-vertebrate Mondo. In addition, while domain names are not generally italicized, we adopt this naming convention to avoid confusion with protein references.

### ChREBP and MondoA are glucose responsive

Current evidence shows both ChREBP and MondoA are glucose responsive, whereby they are mainly located in the cytoplasm under low glucose conditions and have increased nuclear accumulation and transactivation of target genes in high glucose medium [10], [14], [15]. This nuclear translocation and DNA binding is dependent upon the dimerization to obligate partner Mlx, a Max-like transcription factor, which is ubiquitously expressed. Mlx and Mondo proteins contain a C-terminal basic Helix-Loop-Helix-Leucine Zipper (*bHLHZ*) domain responsible for DNA binding and dimerization as well as a dimerization and cytoplasmic localization (*DCD*) domain that must be masked prior to nuclear entry [16], [17]. As shown for MondoA, dimerization through either the *bHLHZ* or *DCD* region is sufficient to block this cytoplasmic retention signal (CRS), but not sufficient for nuclear translocation [16]–[18].

Since MondoA and ChREBP are mainly cytoplasmic proteins, it was surprising to find that trapping them within the nucleus in low glucose conditions was not sufficient to replicate the transactivation potential [19], [20]. Consistent with this, both MondoA and ChREBP are known to shuttle between the cytoplasm and nucleus in both low and high glucose conditions, yet have increased transactivation only under high glucose. In contrast, proteins lacking the N-terminus are able to constitutively transactivate genes in both glucose mediums [16], [21]–[23], indicating additional N-terminal domains within MondoA and ChREBP contribute to their nuclear accumulation and transactivation in response to glucose [16], [21].

### N-terminal conserved regions regulate ChREBP and MondoA activity

MondoA and ChREBP proteins have five Mondo Conserved Regions (*MCRI-V*) in their N-terminus. These have previously been reported as *PADRE1*, *PADRE2*, and *MADRE* [24] as well as a low glucose inhibitory domain (*LID*) which spans *MCRI-IV* and glucose responsive activation conserved element (*GRACE*) which contains *MCRV* [21]. The distances between *MCRII*, *MCRIII*, and *MCRIV* are also conserved, implying they act as a functional module, while the regions linking *MCRI* and *MCRV* vary between MondoA and ChREBP [18]. *MCRII* contains a strong CRM1 dependent nuclear export signal (NES), almost identical to the high affinity LxxLFxxLSV motif. In contrast, *MCRIV* in ChREBP contains a bipartite nuclear localization signal (NLS) that mediates its nuclear entry [15], [25]. Between these two regions *MCRIII* contains a binding motif recognized by the 14-3-3 protein that is involved in ChREBP and MondoA cytoplasmic retention, transactivation, and nuclear export [16], [22], [26]. The functions of *MCRI* and *MCRV* are not as clear, although *MCRI* is necessary for glucose dependent transactivation in ChREBP [27] and *MCRV* is within the *GRACE* region responsible for transactivation [21].

The N-terminal *LID*, containing *MCRI-IV*, possesses a robust repressive mechanism that regulates the strong transactivation region within the *GRACE*. Contrary to prediction, individually deleting or mutating *MCRI*, *II*, *III*, or *IV* also abolishes MondoA or ChREBP transactivation in response to glucose [17], [22], [23]. Hence the *LID* participates in repression in low glucose and activation in high glucose, where no individual *MCR* can sufficiently replicate the glucose response. Moreover, reversing the order of *LID* and *GRACE* regions results in a constitutively active ChREBP protein, indicating its structure and intramolecular contacts are major factors in regulating its function [21].

Deletion and mutation constructs further show each *MCR* seems to have multiple and often opposing function. *MCRI* is necessary for glucose response, since alterations to *MCRI* (ChREBP: Δ1–71, Δ1–58; MondoA: Δ1–100, H78A/H81A/H88A) block transactivation in high glucose, yet mimicking phosphorylation (ChREBP: S56D) enhances it [16], [19], [22], [27]. Likewise, altering the NES in *MCRII* (ChREBP: L89A, F90A; MondoA: F130A, M133A, Δ125–137) mildly enhances transactivation, while other mutations in *MCRII* (ChREBP: L86A/L93A, T85A, L95A, Δ72–99; MondoA: L129A) completely block it [17], [28], [29]. In *MCRIII*, abrogating 14-3-3 protein binding sites (ChREBP: R128A, W130A; MondoA: I166A/W167A/R168A) inhibit transactivation, but so do mutations (ChREBP: N123A, I126A, Δ100–115) that are still capable of interacting with 14-3-3 [17], [19], [22].

Intriguingly, changes within *MCRIV* have even more diverse effects. Some changes (ChREBP: Δ141–197, Δ158–181) likely block the NLS and thus prevent transactivation [23], [25], one change (ChREBP: Δ144–196) reduces transactivation function yet also removes glucose dependent inhibition [22], while another change (MondoA: Y210D/W211D/K212) increases nuclear accumulation and transactivation [17]. While *MCRV* shows no repressive effects in the absence of *MCRI-IV*, changes to it (ChREBP: Y275A/V276A/G277A, L289A/Q290A/P291A; MondoA: Δ282–324) within the full-length sequence lead to an increase in nuclear accumulation and transactivation [17], [19]. Although the cellular conditions, site mutations, and reporter assays in these studies greatly vary, they individually and in combination suggest that the *MCRs* cooperatively repress and activate MondoA and ChREBP function in response to glucose.

### Current models of ChREBP and MondoA glucose response are incomplete

**Figure 1:**
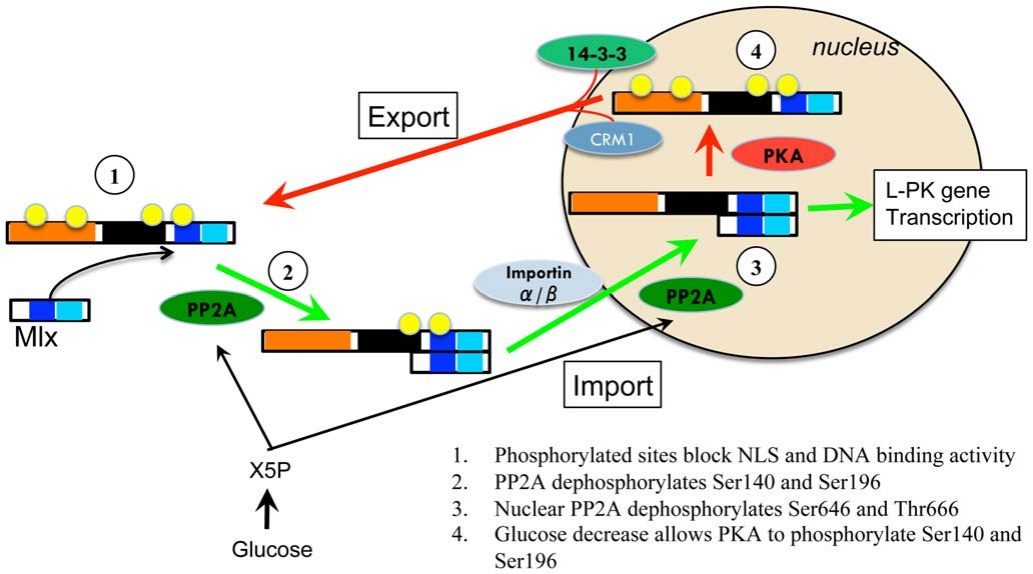
Phosphorylation model depicting ChREBP response to glucose. Image adapted from [29]. 1) In low glucose conditions, sites S140/S196/S626/T666 are phosphorylated and block the NLS and DNA binding activity. 2) Upon glucose stimulation, X5P activates PP2A to dephosphorylate S140/S196 in the cytosol, unblocking the NLS, and allowing ChREBP to enter the nucleus. 3) Nuclear PP2A dephosphorylation of S626/T666 increases DNA binding. 4) Decreased glucose levels increase PKA activity to phosphorylate S140/S196 and shuttle ChREBP back to the cytoplasm.

To properly balance glucose storage and usage, extracellular signals instigate the expression and phosphorylation of proteins involved in the lipogenic pathway. ChREBP contains several such phosphorylation sites [21]. A ChREBP based phosphorylation model postulates that during starvation glucagon increases the concentration of cAMP in hepatocytes, which triggers the phosphorylation of ChREBP by cAMP dependent protein kinase A (PKA) [15]. Phosphorylation of ChREBP site Ser196 causes an adjacent bipartite nuclear localization signal (NLS) in *MCRIV* to be blocked and ChREBP to be sequestered in the cytosol [25]. Conversely, dephosphorylation events mediate a conversion to energy storage rather than usage after a high carbohydrate meal. Increased glucose and thus accelerated glycolytic flux increases the concentration of intermediate metabolite Xylulose-5-phosphate (X5P) within the pentose phosphate shunt, which stimulates protein phosphatase 2A (PP2A) [30]. Cytosolic PP2A mediated dephosphorylation of S196 in ChREBP results in its nuclear localization, while ChREBP DNA binding and transactivation is enhanced by further dephosphorylation of sites S626 and T666 via X5P activated PP2A in the nucleus (Figure 1) [29].

While this simple model is attractive, it is not complete and several issues remain unresolved. Foremost, mimicking the phosphorylation status in ChREBP is not sufficient to activate transcriptional machinery in low glucose [21]. Moreover, MondoA is glucose responsive although it does not contain many of the phosphorylation sites found in ChREBP. In light of recent work, new evidence indicates phosphorylation of glucose by hexokinase to form G6P has a direct impact on the activation of MondoA and ChREBP, although the mechanism is still not known [17], [31]. How G6P is able to promote transactivation within the *GRACE* and override the N-terminal repression imposed by the *LID* region is an important, yet unanswered question. In addition, low glucose repression seems to be independent of a cofactor and is likely a result of protein conformation [23]. Determining the function and interactions of *MCR*s within the N-terminus is of great importance to understanding MondoA and ChREBP glucose response and transactivation of genes involved in glucose metabolism. Since a significant fraction of tumor cells exhibit an increase in glucose metabolism and direct glucose into *de novo* lipogenesis [32], [33], understanding the specific roles of MondoA and ChREBP in glucose regulation can directly affect the treatment of such diseases.

Herein, we synthesize the current knowledge of Mondo family proteins and domains into a cohesive, accurate, and generalized model to address Mondo activation in response to glucose. First, we hypothesize that MondoA and ChREBP domains function analogously and defend that their overall conservation implies similar structure and function among Mondo proteins. Second, we identify a novel domain and propose it is involved in sensing changing glucose levels and altering Mondo transactivation potential. Finally, we form a unified model based on current data that explains MondoA and ChREBP subcellular localization and transactivation in response to glucose. Together, this information forms a more complete picture for how Mondo proteins, in general, respond to elevated glucose levels and creates a series of testable hypothesis, which can be experimentally validated to refine our understanding of glucose metabolism.

## Results

### *MCRI-V*, *bHLHZ*, and *DCD* domains are conserved among Mondo protein sequences

According to previous reports [16], [18], the similarity within Mondo protein sequences is largely contained within the *MCRI-V*, *bHLHZ*, and *DCD* domains. However, the BLOCKS and MEME approaches in these papers were used to simply present delimited regions of increased conservation without commenting on the constraints or functional contribution of each residue. Here we include orthologous Mondo proteins from several ancient and intermediate lineages, such as the Placazoa *Trichoplax adhaerhens* and Cnidaria *Nematostella vectensis* to help explicate the evolution of Mondo conserved domains as well as the imposed functional constraints.

**Figure 2:**
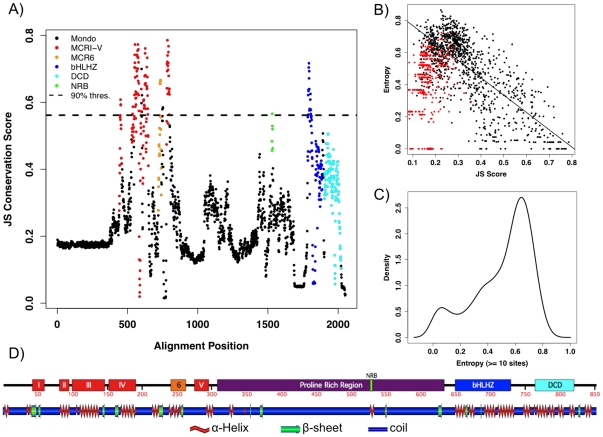
Mondo sequence and structure conservation. **A**) **JS Conservation Score.** All Mondo sequences were used to construct an alignment of homologous sites. Black dots represent alignment columns, while sites within domains are colored: red: *MCRI-V*, orange: Myc box II-like (*MCR6*), green: nuclear receptor box, blue: basic helix-loop-helix-zipper, cyan: *DCD*. The dashed line sets the 90% threshold for JS scores **B**) **JS and Entropy Comparison.** red: sites with less than 10 residues, black at least 10 residues, where linear regression was performed on the latter with intercept = 0.8745, slope = −1.0803, r^2^ = 0.55467. **C**) **Entropy Distribution.** Distribution of entropy values for sites with at least 10 residues **D**) **Domains and Secondary Structure.** Consensus secondary structure for ChREBP shown alongside its sequence domains.

To more precisely identify and quantify the conservation within Mondo family proteins among diverse organisms, we created a multiple sequence alignment consisting of numerous species sampled across the animal kingdom (see Methods). This allowed us to directly observe the conservation of each alignment column through the Jenson-Shannon Divergence (JS) score (Figure 2), which rates each site by an autocorrelated conservation value [34]. Since conservation is a powerful predictor for detecting functional sites, sites within more conserved regions have higher JS values and are thus more likely to affect protein function (Figure 2a). Similarly, entropy (H) measures the amount of information or variability within an alignment column where conserved sites have low entropy values. As expected, sites within the *MCR*, *bHLHZ*, or *DCD* regions are highly conserved and have correspondingly high JS and low H values.

However, the relationship between JS and H is nonlinear due to several autapomorphies within the full sequence alignment (Figure 2b). In these cases, sequence specific insertions or poor prediction of exon boundaries for unannotated sequences create alignment columns with just a single or few residues. By removing alignment positions with less than ten residues, we were able to recover the correlation between entropy and JS scores (r^2^ = 0.55), as well as reveal two peaks in entropy values (Figure 2c). From this reduced dataset, 127 (11.6%) sites are considered highly conserved with H<2.0, while most other sites are variable. Since JS values are scored using an adjacency window, the JS distribution is smoothed to form a single peak and there is no clear delineation of conserved and variable sites (data not shown). In accordance with entropy values, setting an arbitrary 90% threshold (JS>0.5597) shows the most conserved sites are within the *MCR* and *bHLHZ* regions (Figure 2a).

**Figure 3:**
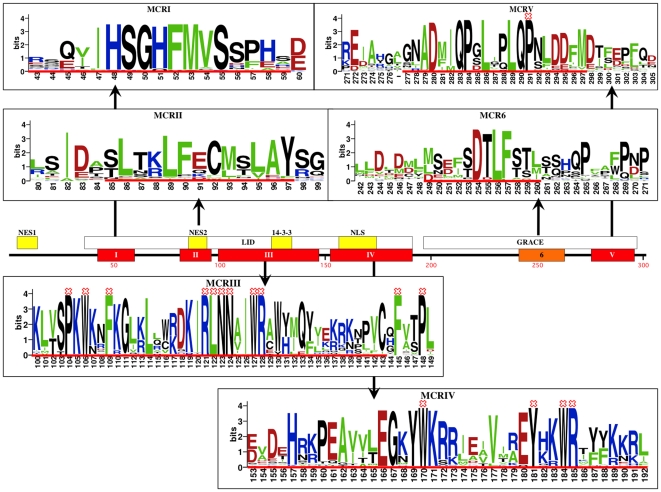
Mondo conserved regions. MondoA and ChREBP have five previously defined and uniquely conserved regions, i.e. *MCRI-V*. These have been grouped into the *LID* and *GRACE* regions in ChREBP, and annotated for nuclear export signals (NES1, NES2), α-helix necessary for 14-3-3 binding, and a bipartite nuclear localization signal. These domains, along with newly identified *MCR6*, are highly conserved among Mondo sequences, with Mondo invariant positions marked with a red ‘X’. Weblogos depicting the particularly conserved sites and regions were created using the full Mondo alignment, with the previously defined MCR regions designated by a red line. We use the red line in MCR6 to accentuate the 12 residues with increased conservation in this region. Amino acids are colored so basic (HKR) residues are blue, acidic (DE) are red, and hydrophobic (AVLIFM) are green. Numbering is according to human ChREBP sequence.

High JS scores were also observed for two new and potentially important regions. The first region, which we name Mondo Conserved Region 6 (*MCR6*), was previously reported as a MBII-like region located between *MCRIV* and *MCRV* [18]. However, the MBII-like region designated by the previous alignment showed little similarity in amino acid compositition. From our dataset, we were able to improve the alignment and identify a highly conserved [ST]DTLF[ST] motif, where [ST] indicates either a serine or threonine. The conservation of *MCR6* residues, as well as *MCRI-V*, is depicted by the weblogos in Figure 3, where larger letters indicate more conserved sites. Based on the distribution of amino acids, we propose *MCR6* be defined by the 12 residue sequence signature [MLD][SNED][EDML] [FIM][ST]DTLF[ST][STM][LTI].

### Mondo proteins exhibit divergent domains

**Figure 4:**
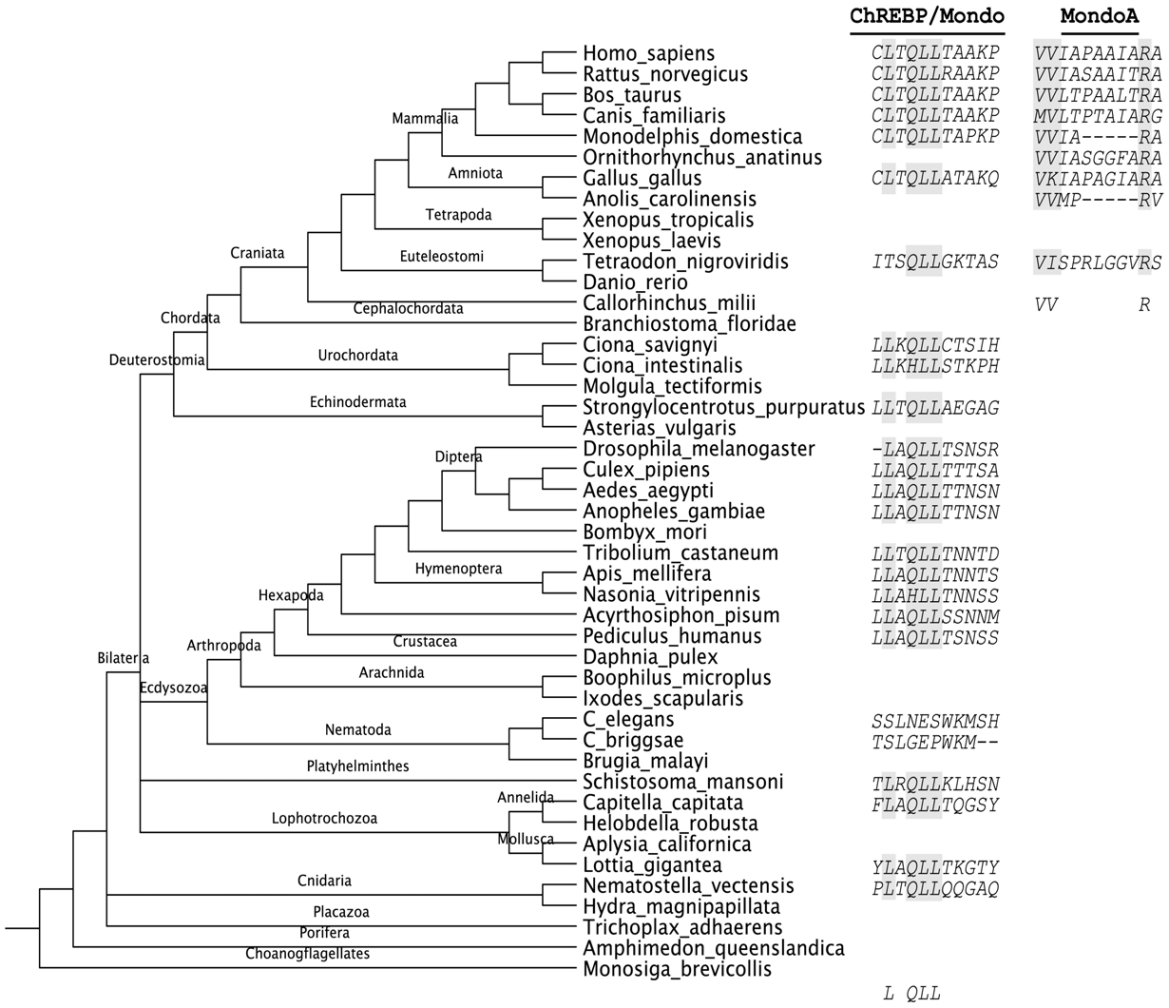
Nuclear receptor box conservation. A LxQLLT motif is largely conserved among animals. Since we could not obtain the full sequence of all sampled species (shown in the species tree), many display alignment gaps, which do not necessarily indicate they lack the putative *NRB*. However, MondoA in vertebrates exhibits a divergent sequence and lacks the *NRB*.

JS scores also revealed a novel LxQLLT motif located within the central region of ChREBP and non-vertebrate Mondo protein sequences, but not MondoA (Figure 4). This sequence conforms to the LxxLL nuclear receptor box (*NRB*) signature that participates in the ligand dependent activation of nuclear receptors. *NRB*s are found within nuclear receptor coactivators such as the SRC-1 family of proteins (pfam ID: PF08832), which typically have multiple repeats of this motif, each sufficient for ligand interaction with several nuclear receptors [35]. Non-vertebrate Mondo and ChREBP proteins only contain one putative *NRB*. Interestingly, ChREBP and nuclear receptor HNF4α have adjacent recognition sequences in the promoter sequence of liver pyruvate kinase (L-PK) [9], [36]–[38]. Full activation of the L-PK gene requires both ChREBP and HNF4α [37], and ChREBP:HNF4α:CBP is recruited as a complex to the L-PK promoter region in a glucose dependent manner [39]. Taking this into consideration, it is reasonable to assume that the ChREBP *NRB* is capable of activating HNF4α.

Conversely, MondoA, but not ChREBP, localizes specifically to the outer mitochondrial membrane (OMM) when in the cytosol [10]. Mitochondria import stimulating factor (MSF) was identified as a mitochondrial chaperone and is a member of the 14-3-3 protein family [40]. Chaperone proteins transport cargo proteins to the mitochondria that contain a presequence located in the distal N-terminus. Generally, mitochondrial surface proteins cleave this preprotein sequence, which allows the mature protein to enter through the mitochondrial membrane. However, some OMM proteins have a distal N-terminal, preprotein sequence that is not cleaved. In these few cases, this sequence is used for mitochondrial targeting, but not cleavage or import [41].

We find that MondoA, but not ChREBP or non-vertebrate Mondo proteins, are predicted to contain mitochondrial targeting peptides within the first 42 residues, as specified by the program TargetP [42]. MondoA is not known to enter the mitochondria [10] or predicted to contain a transmembrane region that inserts into the OMM. Hence we propose the N-terminus sequence of MondoA induces mitochondrial transport via 14-3-3, where it interacts with receptors located on the OMM. This novel function may further contribute to glucose sensing and regulation in skeletal muscle, where MondoA is preferentially expressed.

### The importance of *MCR* and *DCD* invariant positions

**Figure 5:**
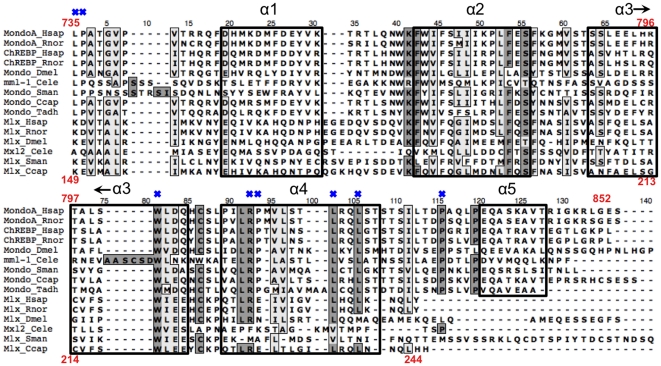
Mondo and Mlx *WMC/DCD* alignment. *DCD* region of Mondo and Mlx sequences from *Homo sapiens* (Hsap), *Rattus norvegicus* (Rnor), *Drosophila melanogaster* (Dmel), *Caenorhabditis elegans* (Cele), *Capitella capitata* (Ccap), and *Trichoplax adhaerens* (Tadh). Red numbering on top corresponds to human ChREBP position, while the bottom represents the Mlx numbering. Sites with >75% identity or chemical similarity are shaded dark and light gray respectively, while the five (four) predicted alpha helices for MondoA and ChREBP (Mlx) are boxed. Mondo invariant positions are marked with a blue ‘X’.

By isolating columns with zero entropy and hence no variation, we identify 24 invariant sites within the Mondo sequence alignment, all of which are contained within the *MCR* and *DCD* regions (see Figures 3 and 5). We hypothesize that these sites are crucial for proper function of Mondo family proteins and find that many have been reported as essential for MondoA or ChREBP interactions or transactivation.

*MCRIII* contains two groupings of invariant residues P104/W106/F109 and R121/L122/N123/N124/W127/R128 (human ChREBP numbering used throughout, except when directly referencing MondoA). Accordingly, the α-helix spanning ChREBP sites 116–135 is essential for 14-3-3 binding as is R128A [22], suggesting the invariant RLNN and WR residues are involved in 14-3-3 interactions. However an N123A mutation demonstrates it not necessary for 14-3-3 binding, but is essential for transactivation [19]. In comparison, mutation to MondoA sites **P144A**/K145A/**W146A** (human MondoA numbering, invariant sites in bold) did not affect 14-3-3 binding and no other phenotypic variations were reported [16]. However, we found that a serine or threonine immediately precedes P104 in all sequences, indicating this may be an important phosphorylation site for Mondo proteins.

Sites F145 and P148 are also invariant, yet have not been previously included in a specific *MCR* sequence. These residues (bold) are within a conserved [KR]x[KRN][NSTP][PLIV][VFI][CIV]x**F**[AVI][STV]**P**[LIV] motif that is located directly downstream of *MCRIII* (underlined). With the exception of upstream insertions within tunicate *Molgula tectiformis* (KILRRYGY), and nematodes *C. elegans* (KKQP) and *Brugia malayi* (RPDKD), this conserved region is contiguous with the remainder of *MCRIII* and thus we include these additional sites within *MCRIII* (Figure 3). As before, the prevalence of serine and threonine residues before P148 suggests a putative phosphorylation site in Mondo family proteins, with the exception of orthologous MML-1 proteins in nematodes, which have a valine instead.

*MCRIV* sites W170/Y181/W184/R185 are also invariant, along with P291 of *MCRV*. Analogous to ChREBP sites Y169/W170/K171, alanine mutations of MondoA sites Y211/**W212**/K213 resulted in nuclear accumulation in low and high glucose as well as three-fold induction of TXNIP reporter gene in L6 myoblasts [17]. Similar results were observed for L289A/Q290A/**P291A** mutation in ChREBP with two-fold ACC gene reporter expression in 832/13 cells [19]. Hence these sites are likely involved in repression of Mondo family proteins.

The remaining eight invariant positions are within the *DCD* region, represented by ChREBP sites L735, P736, W801, R812, P813, L819, L822, and P832. While their function is unknown, sites L735/P736 are located directly after the *bHLHZ* and may be important for correctly orienting the *DCD* domain. The conservation of this region is addressed in later sections.

Surprisingly, *MCRI*, *MCRII*, and the *bHLHZ* region lack invariant residues. However, high JS scores indicate these regions as well as others within *MCRIII* and *MCRIV* are still functionally conserved among species. For example, divergence of the predicted protein sequence in beetle *Tribolium castaneum* (XP_973749.2) prevents the identification of otherwise invariant residues **HSG**x**FM**x**S** within *MCRI*, where bold letters are conserved and x represents a variable site. *MCRII* in *Tribolium* is also not conserved, suggesting its N-terminal region is divergent or incorrectly identified. Regardless, most *MCRII* site variability arises from divergence in nematodes and other more distantly related species, which may indicate changes in selective pressure in Arthropoda and Deuterostoma lineages. In contrast, no single sequence is responsible for *bHLHZ* variability, although it appears that nematode, ghost shark *Callorhinchus milii*, and sea squirt *Ciona intestinalis* often differ at otherwise conserved sites. Conservation of the *bHLHZ* is addressed in detail in [12].

### N- and C-terminal regions of Mondo family proteins have conserved secondary structure

Considering the extent of sequence conservation among species, we further hypothesize Mondo proteins exhibit similarity in higher order structures. As expected, we found secondary structure predictions of ChREBP, MondoA and non-vertebrate Mondo proteins are comparable, and the majority of their protein sequences are random coil with several α-helices and intermittent β-sheets (Figure 2d, Fig. S1). Predictably, the α-helices and β-sheets overlap the *MCR*, *bHLHZ*, and *DCD* conserved regions described above, as well as *MCR6* and the *NRB* in non-vertebrate Mondo and ChREBP sequences. This implies the conserved residues are similarly orientated within the domains and Mondo family proteins are composed of the same structural elements.

**Figure 6:**
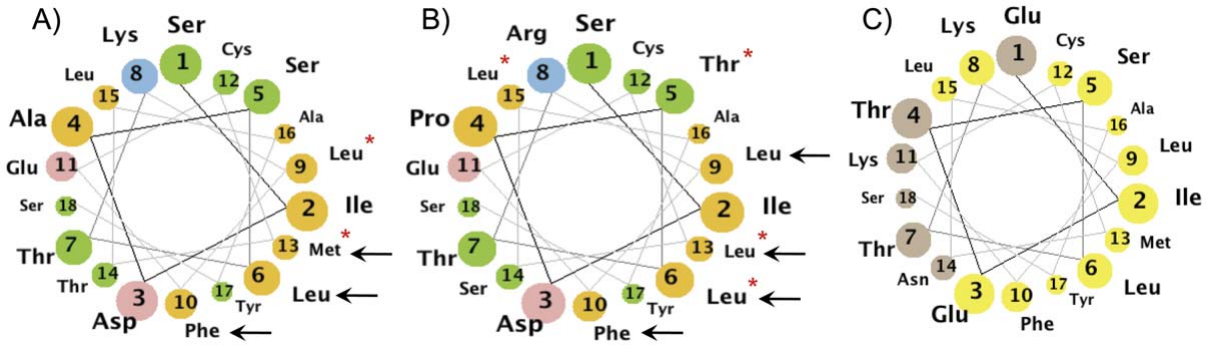
*MCRII* helical wheel. **A**) MondoA sites 121–138, **B**) ChREBP sites 81–98. Helical numbering is according to position within *MCRII* and represented by decreasing circle sizes. Black arrows point to sites indicated as essential for NES and red asterisks mark those necessary for glucose responsive transactivation. Color scheme: blue-basic, pink-acidic, orange-nonpolar, green-polar, uncharged. **C**) *Drosophila* sequence. Yellow circles have at least 75% chemical identity among all Mondo sequences.

We also predict secondary structure plays a role in maintaining the function of these conserved Mondo domains. In support of this premise, experiments show the α-helices comprising the *bHLHZ* and *DCD* domains are necessary for basic Mondo protein function, e.g. DNA binding, dimerization and subcellular localization [16]–[18]. Likewise, the three α-helices within *MCRII*, *MCRIII*, and *MCRIV* correspond to a NES, 14-3-3 binding region, and NLS respectively and are critical for proper function [29]. In particular, *MCRII* residues have been found to be independently essential for transactivation in addition to CRM1 dependent nuclear export [19]. Projecting the residues of *MCRII* onto a helical wheel, we find the residues necessary for these functions are more highly conserved and located on the same side of the α-helix (Figure 6). Hence the relative orientation of these residues possibly creates a surface for competitive interaction mediating a transition in functions.

### Evidence for a CRS in *MCRIV*

MondoA:Mlx and ChREBP:Mlx heterodimers actively shuttle between the nucleus and cytoplasm, indicating that increased nuclear accumulation in response to glucose is not simply the result of nuclear targeting (Table S1). In fact, all the *MCR*s affect the subcellular localization of ChREBP and MondoA. Blocking the *MCRII* NES in either MondoA (M133A, F130A, MondoAΔ125–137) or ChREBP (ChREBPΔ86–95, ChREBPΔ72–99, L86A/L93A, L89A, F90A) results in nuclear accumulation in either low or high glucose conditions [17], [19], [22], [28], [29]. Likewise, altering the *MCRIV* NLS in ChREBP (ChREBPΔ158–173, ChREBPΔ158–173, ChREBPΔ168–190) results in cytoplasmic retention [25], [28], [29]. However, MondoA triple mutant Y211A/W212A/K213A, which overlaps the latter portion of the bipartite NLS in ChREBP, results in MondoA nuclear localization in low and high glucose in L6 myoblasts [17]. In addition, C-terminal sequences, optionally including *MCRV* and *MCR6*, result in nuclear accumulation for both MondoA [16] and ChREBP [21], [29]. However, the inclusion of residues 224–273 in MondoA resulted in a cytoplasmic shift with most cells having equal nuclear and cytoplasmic amounts, while a MondoA mutant containing the full *MCRIV* region (MondoA:182–919) slightly reversed this effect with most cells being nuclear [16]. This suggests that MondoA *MCRIV* has opposing roles in nuclear localization.

It has been suggested that MondoA *MCRIV* contains a CRS [18] and truncation mutants indicate it is located within the latter half of *MCRIV*. The bipartite NLS in ChREBP *MCRIV* is only partially conserved in some MondoA sequences, due to a single arginine to serine mutation (MondoA:R213S) arising prior to the divergence of canines. Interestingly, the basic residues within the first portion of the NLS are conserved in MondoA, but variable in non-vertebrates, suggesting that the NLS may be weak, dispensable, or nonexistent in these proteins. As such, fusing *MCRIV* of MondoA to a heterologous NLS resulted in complete cytoplasmic localization [18]. This is independent of 14-3-3, which binds to *MCRIII* and was previously implicated in cytoplasmic localization. Together this data implies MondoA:224–273 contains a strong CRS. We found this region is similarly conserved among Mondo family proteins, with sequence signature VxxE**Y**[KH]K**WR**x[FY][FY][KR], where x represents a variable site and bold letters are invariable among Mondo sequences. Due to this conservation, we hypothesize that ChREBP and non-vertebrate Mondo proteins also contain a CRS within *MCRIV*.

Directly downstream of *MCRIV*, site S196 dephosphorylation results in the nuclear accumulation of ChREBP in low and high glucose [25]. Since MondoA and non-vertebrate Mondo proteins lack this phosphorylation site but have glucose-responsive subcellular localization, we anticipate the putative phosphorylation site 147-[TS]P-148 between *MCRIII* and *MCRIV* (ChREBP numbering) may be involved, as it is found in almost all Mondo family proteins and phosphorylated in high glucose for ChREBP triple mutant S196A/S626A/T666A [27].

### *DCD/WMC* is conserved among Mlx and Mondo family proteins

For MondoA, and presumably ChREBP, to enter the nucleus, dimerization with Mlx must first occur. This is due to a cytoplasmic retention signal (CRS) located within the *DCD*, which is directly downstream of the *bHLHZ* domain [16], [17]. The *DCD* region provides an additional and independent interaction interface between Mondo family and Mlx proteins, which masks the CRS and allows for nuclear entry. While most of our understanding regarding this region is based on MondoA mutations, observations concerning the homologous and extended sequence WBSCR14-Mlx C-tail (*WMC*) region of ChREBP provide similar results [26], [43]. Still, little is known about how the *DCD/WMC* region acts as a CRS, dimerizes, or differs between Mondo and Mlx proteins.

To determine which residues within the *DCD/WMC* potentially contribute to its structure or function, we compared Mondo and Mlx protein sequences using multiple entropy measures (see Methods). From the *DCD/WMC* alignment columns (Figure 5) containing more than three residues, sites K41, F42, W81, L91, and L102 are nearly invariant across all Mlx and Mondo sequences with entropy less than 0.1 (H<0.1), while columns 5, 6, 13, 21, 41, 42, 44, 55, 56, 60, 81, 82, 83, 86, 91, 96, and 102 (*DCD/WMC* alignment numbering) display conservation with functional entropy less than 0.1 (H_FG_<0.1) (Figure S2). As expected, sites with H<0.1 also have H_FG_<0.1. This is consistent with experimental evidence, which show residues K41, F42, S54, and F56 of MondoA and Mlx are important determinants of heterodimerization [16]. Compared to the Mondo invariant sites described previously, only W81 is invariant in both Mondo and Mlx, although L91 is conserved in all but the nematode sequences.

Based on the *DCD/WMC* conservation, our results disagree with the claim that *C. elegans* MML-1 lacks a *DCD* region [44]. We find that *C. elegans* MML-1 is conserved at 10 (58.8%) of the 17 functionally constrained sites as well as the eight invariant Mondo residues. Moreover, the *DCD/WMC* region of MML-1 is 46.7% similar and 21.3% identical to mosquito *Culex pipiens*, while nematode Mlx homolog Mxl-2 is 40% similar and 16.2% identical to the Mlx *DCD/WMC* sequence in beetle *Tribolium casteum*. Hence, we assert that the *DCD/WMC* region is intact in *C. elegans* MML-1 and Mxl-2 proteins. Since these nematode sequences contain *MCR* and *DCD* domains that define Mondo and Mlx proteins, we further defend that MML-1 (myc- and mondo-like 1) is within the Mondo family and Mxl-2 is an ortholog of Mlx. This corroborates with the phylogenetic classification of their bHLHZ sequences [12].

### *DCD/WMC* structure forms an α-helix bundle

To determine the importance and potential interactions among conserved sites within the *DCD/WMC*, we predicted the higher order structures of this region. Secondary structure predictions of the *DCD/WMC* for MondoA, ChREBP, and non-vertebrate Mondo proteins identifies five α-helices, while only four were found for Mlx sequences (Figure 5). Previously, just the *DCD* region was considered in structure prediction of ChREBP and a zipper like tertiary structure was assumed [45]. However, by including the entire *WMC* region, the powerful 3-D structure software Rosetta predicts the ChREBP *DCD/WMC* model assumes a cyclin-like confirmation with five grouped α-helices, Figure S3a [46]. This predicted configuration forms a groove flanked by hydrophobic residues in alpha helices 1, 2, 3, and 4 designated by alignment sites **21**, 25, and 29 of α1, **44**, 47, 48, 49, 52 of α2, 65, 68, 73, and **82** of α3, and 88, **91**, 95, **96**, **102** and 105 of α4, where functionally conserved residues (H_FG_<0.1) are in bold.

This interior region also displays increased conservation according to both entropy and Consurf estimates (Fig. S3b). The program Consurf estimates the evolutionary rate of each site by comparing homologous sequences and similar protein structures [47]. Consurf predicts ChREBP residues V6, K41, F42, S55, W81, L88, and L102 (*DCD/WMC* alignment numbering) have high conservation scores and are likely functionally important. Besides L88, these positions have low functional entropy for all Mondo and Mlx sequences, suggesting a common function.

The *DCD/WMC* of Mlx and Mondo family proteins show clear similarity, although we anticipate protein distinctions likely affect their tertiary conformation. First, our alignments show the *DCD/WMC* region of Mlx abuts the 21-residue zipper region, while the zipper and a linker region of Mondo sequences together extend for 35 residues before the *DCD/WMC* begins. In addition, Mondo invariant sites L735/P736 are alternatively conserved for charged residues (lysine and either aspartate or glutamate) in Mlx, which may affect the *DCD/WMC* orientation. Moreover, helix 5 shows considerable variability among the Mondo sequences, and may not be directly involved in protein-protein interactions, as it is completely lost in most Mlx sequences. These differences may restrict interaction between *DCD/WMC* regions and factor in the prevention of MondoA and Mlx homodimerization [16].

### Mondo proteins have disparate Proline and Glutamine Rich Regions

In contrast to the structured N- and C-terminus, the central region of Mondo proteins is mainly composed of random coil. Both MondoA and ChREBP proteins contain a proline rich region (*PRR*) within their proximal region that is retained among most vertebrates. However, we were unable to find any identifiable stretch of homology between MondoA and ChREBP *PRR*s and the *PRR* is not found within any non-vertebrate species. Instead, most non-vertebrates contain a glutamine rich region (*GRR*) (Table 1). The prevalence and length of these low complexity regions suggests the central region contains an imprecise function, such as indiscriminate scaffolding regions as seen in other *PRR* and *GRR* containing proteins [48], [49] and may contribute to Mondo transactivation of target genes.

**Table 1:** Proline and Glutamine Rich Region.

		Proline	Glutamine	Neither	Missing
Vertebrates		16	0	1	3
Non-vertebrates		0	10	6	10
Length	*mean*	355.75	543	462.14	
	*sd*	48.9	173.4	104.3	

Existence of Proline Rich and Glutamine Rich Regions in the proximal domain of Mondo sequences as predicted by ScanProsite. Neither indicates the central region is intact, yet ScanProsite did not identify a *PRR* or *GRR* region. Missing denotes sequences where the central region was only partially or not recovered. Length is calculated by the number of amino acids between the *MCRV* and *bHLHZ* of full length sequences.

### *MCR6* involvement in Glucose Dependent Activation

Recent evidence shows that MondoA and ChREBP activation is dependent upon glucose phosphorylation by hexokinase, which metabolizes glucose to form glucose-6-phosphate (G6P) [17], [20], [31]. Induction of 2-deoxyglucose (2-DG), which is a glucose analog that can be phosphorylated but not further metabolized, promotes MondoA nuclear accumulation, increases promoter occupancy and recruits histone H3 acetyltransferase thereby activating gene transcription [17]. Similarly, 2-DG dose dependently increased the transactivation ability of Gal4-ChREBP, while hexokinase inhibitor d-mannoheptulose and glycolytic enzymes PFK1 and PFK2 decreased ChREBP activity [31]. This suggests that MondoA and ChREBP activation is directly invoked by glucose phosphorylation. Moreover, the N-terminus of Drosophila ortholog dMio activates a luciferase reporter comparable to Gal4-ChREBP levels in a glucose responsive manner [21]. Domain swapping of the LID region of ChREBP with that of either MondoA or dMio resulted in a strong glucose response, suggesting that the LID and GRACE regions are interchangeable among homologs and Mondo proteins, in general, are glucose responsive. As such, we hypothesize regulation of Mondo family proteins is expected to occur through a G6P mediated signaling cascade, direct binding of G6P to an allosteric mechanism, or both.

**Figure 7:**
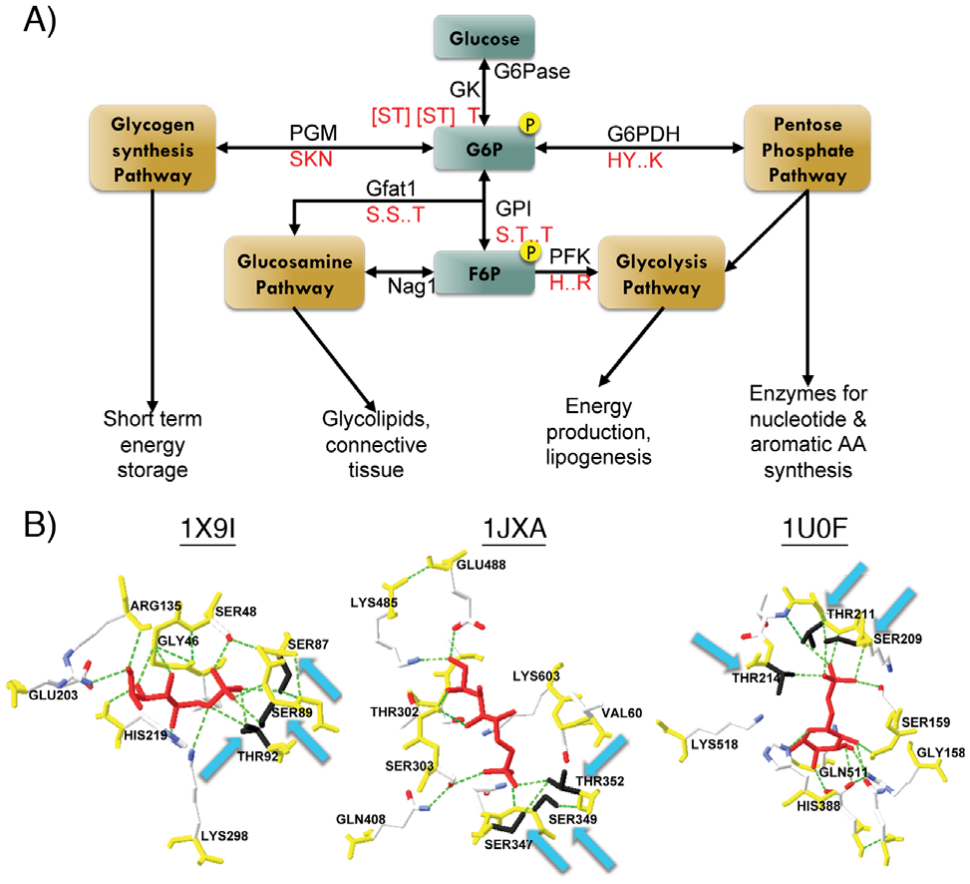
G6P binding region. **A**) Glucose metabolism pathways. Glucose is phosphorylated in the liver by GK to form G6P. G6P can then enter the pentose phosphate pathway by interacting with G6PDH, the glycogen synthesis pathway by binding to PGM, or form F6P by GPI isomerization. Residues involved in these interactions are shown in red, with dots indicating nonbinding sites within a linear sequence and spaces denoting larger linear distances. **B**) G6P interacting protein structures. The structures for GPI in Rat (1U0F), phospho-glucose/phospho-mannose protein in archaea (1X9I), and GlmS in *E. coli* (1JXA) have been crystallized. The backbone of residues within 5 Angstrom of G6P (red) are yellow and hydrogen bonds are shown by a green dashed line. We indicate the residues conforming to the G6P recognition motif with blue arrows and color the side chains black.

To investigate the presence of an allosteric G6P binding region within Mondo proteins, we first examined the binding region of known G6P interactors (Figure 7), i.e. glucokinase (GK), hexokinase (HKI-III), G6P phosphatase (G6Pase), phosphoglucose mutase (PGM), glucose phosphate isomerase (GPI), G6P dehydrogenase (G6PDH), and glutamine:fructose-6-phosphate amidotransferase (human: Gfat1, *E.coli*: Glms). Since glucose is essential among prokaryotes and eukaryotes, the enzymes and binding regions involved in glucose metabolism are highly conserved. Interestingly, we find the G6P binding region is similar among GK, GPI, and Gfat1, with serine and threonine residues forming hydrogen bonds with the 6-phosphate molecule (Figure 7b). Moreover, the phosphate recognizing residues of GPI and Gfat1 are in close proximity in the linear sequence, forming an Sx[ST]xxT motif, where x indicates a residue not involved in 6-phosphate recognition. This is distinct from G6PDH and PGM, which have HYxxK and SKN motifs, respectively.

We propose G6P binds to Mondo proteins within the highly conserved *MCR6* region, which contains an Sx[ST]xx[ST] motif similar to that found in GPI and Gfat1. Our alignments show MondoA consists of residues 281-**S**D**T**LF**S**-287, while ChREBP contains a 253-**S**D**T**LF**T**-258 motif. This putative G6P recognition motif is also preserved in non-vertebrate Mondo sequences, where serine and threonine are likely to interchangeably form hydrogen bonds with the 6-phosphate molecule. We predict this motif is associated with recognizing the phosphate group of G6P, which is consistent with the correlation between MondoA and ChREBP activation and glucose phosphorylation.

While the strict conservation of Sx[ST]xx[ST] within *MCR6* among animals is evidence for its functional importance among Mondo proteins, this short motif has low specificity and is predicted to occur in several sequence locations. By plotting the location of each Sx[ST]xx[ST] motif for each Mondo sequence (Fig. S4), we find that this motif is not distinctly conserved elsewhere in the alignment, suggesting these residues in *MCR6* are functionally constrained.

In addition, *MCR6* is located within the GRACE region, which is sufficient for ChREBP transactivation [21]. Interestingly, mutations to the only other conserved domain within the ChREBP GRACE region, *MCRV*, show an increase in transactivation [19]. ChREBP:299–645, which is downstream of the GRACE region and encompasses the Proline Rich Region, is also sufficient for transactivation. Meanwhile, ChREBP:197–479, which overlaps the GRACE and PRR, shows a significant increase in fold activation of a luciferase reporter, suggesting a synergy between these domains [21]. This is compatible with the TAD domain found in MondoA 322–445 [11], which overlaps its PRR.

We hypothesize *MCR6* of the *GRACE* region harbors a TAD that contributes to the recruitment of coactivators such as CBP/p300, which are known to interact with ChREBP [39]. To test this, we searched the entire sequence of each Mondo protein for the nine amino acid transactivation domain (9aa TAD) signature that is recognized by coactivators TAF9, MED15, CBP, and p300 [50]. Although individual sequences displayed multiple hits using the 9aa TAD regular expression (see Methods), the only common occurrence was in *MCR6* where we observed two overlapping 9aa TAD motifs. ChREBP was restricted to motif 1 (ChREBP:250-SDIS**DTLF**T-258), while MondoA and Mondo sequences also matched motif 2 (MondoA:283-**DTLF**STLSS-291); conserved sites within the overlapping regions are in bold and underlined. Of the 34 sequences in our dataset containing *MCR6*, nineteen contained both motif 1 and 2, five only had motif 2, eight only had motif 1, trematode *Schistosoma mansoni* matched an intermediate sequence, and sea anenome *Nematostella vectensis* matched neither. Although there was no clear preference for either motif, we propose the TAD is located in *MCR6*, and consider that the presence of multiple TAD motifs within this region may provide variable specificity for binding cofactors.

### *LID* and *GRACE* regions have intramolecular contacts in N-terminal Predicted Structure

The *LID* region, containing *MCRI-IV*, is necessary to repress transactivation in low glucose conditions and promote transactivation in high glucose conditions [21]. However, how the *MCRI-IV* domains individually and cooperatively operate is not clear. To better understand how *MCRI-IV* switches between repressive and activating functions, we predicted the protein structure for MondoA and ChREBP N-terminal sequences.

**Figure 8:**
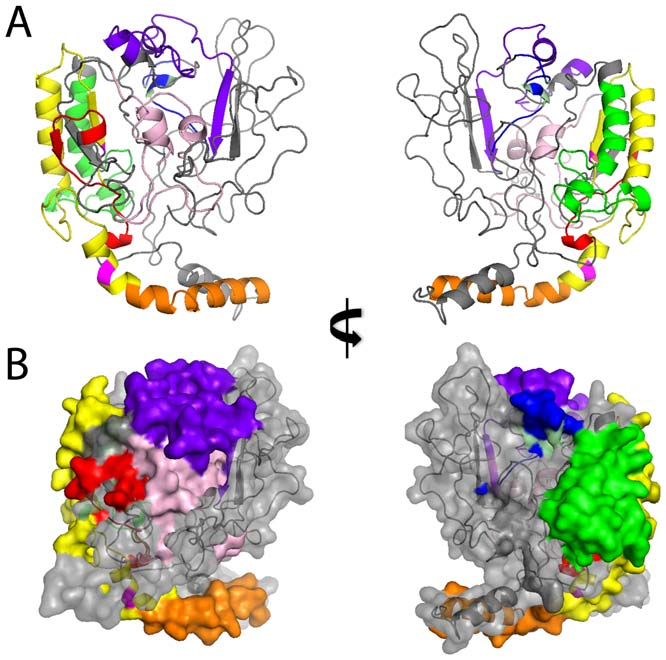
MondoA N-terminus structure. Predicted structure for MondoA:1–490. **A**) Ribbon structure. **B**) Filled structure. *MCRI* is red, *MCRII* is orange, *MCRIII* is yellow, *MCRIV* is green, *MCR6* is blue, and *MCRV* is purple. In addition, the first 42 residues potentially targeting MondoA to the OMM are light pink, and putative phosphorylation sites S143 and T187 are magenta, and the serine and threonine residues of *MCR6* are pale green. Left and right images are rotated 180 degrees.

**Figure 9:**
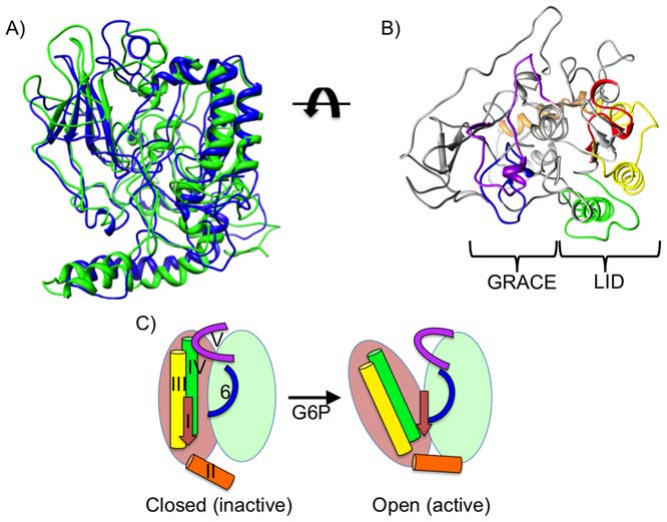
*LID* and *GRACE* interaction. **A**) MondoA (green) and ChREBP (blue) overlay of N-terminal predicted structure. **B**) Topical view of MondoA:1–490 ribbon structure. *MCRV* and *MCR6* are part of the *GRACE* region, while the *LID* includes *MCRI-IV*. *MCR* domains are colored as in Figure 8. **C**) Predicted allosteric affect of G6P binding to *MCR6*. *MCRII* and *MCRIII* release from *MCRV*, while *MCRI* and *MCRII* lock the “open” conformation to separate the *LID* and *GRACE* regions and support transactivation.

From the sequence and secondary structure predictions of 3D-Jury, the N-terminus of MondoA was most similar to Estrone Sulfatase (ES, PDB ID: 1p49) (Figure 8) and also showed a likeness to similar sulfatase structures (PDB ID: 1auk, 1fsu). As expected, the N-terminus of ChREBP also shows structural similarity to 1p49 and resembles the MondoA conformation (Figure 9a).

The putative MondoA and ChREBP protein structures are compatible with the accessibility of their known domains. The protruding α-helices in MondoA and ChREBP correspond to *MCRII* and its CRM1 dependent NES in the predicted structure (Figure 8, orange). This is concordant with the CRM1-SNUPN structure, where the NES of SNUPN forms an extended amphipathic α-helix that protrudes away from the rest of the molecule and binds a hydrophobic groove in CRM1 [51]. The exposure of *MCRIII* (Figure 8, yellow) also allows for its α-helix to interact with known binding partner 14-3-3. The orientation of *MCRIII* and *MCRIV* (Figure 8, green) α-helices closely position S140 and S196 in ChREBP, so they are both situated near *MCRV* (Figure 8, purple; Fig. S5). This conformation agrees with evidence implicating S196 and S140 phosphorylation affects nuclear accumulation and 14-3-3 interaction [29] as well as the interaction model hypothesized by Davies et al. [23].

The placement of *MCRV* near the ends of *MCRI* (Figure 8, red), *MCRIII*, and *MCRIV* allows for interaction among these domains and corresponds to the proposed linkage between *LID* and *GRACE* regions mediated by multiple contacts with *MCRV* [21], [23]. *MCR6* (Figure 8, blue) is adjacent to *MCRIV* and may also have a binding interface. Considering the potential role of *MCR6* in G6P binding and transactivation, this interaction may affect the glucose response, as seen for proteins with *MCRIV* deletions that lack glucose dependent regulation [22]. Viewing the predicted structure from the top (Figure 9), it is easy to see how the *LID* can contact and possibly release from the *GRACE* region to conditionally block the binding of coactivators and regulate the transactivation of target genes.

## Discussion

Conservation in sequence, domains, and glucose response for MondoA and ChREBP proteins suggest they are mechanistically similar. Based on the elevated JS conservation scores and persistence of secondary structures across sequences, the distal regions of Mondo proteins are likely to exhibit similar structure and function. The presence of *MCRI-V*, *MCR6*, *bHLHZ*, and *WMC/DCD* regions in diverse organisms dates the origin of these regions to as early as the divergence of cnidarians around 600 million years ago [52]. Moreover, conservation of Mondo proteins and domains throughout animal evolution suggests the glucose responsive transactivation observed in MondoA and ChREBP has been preserved as well. Similar to the explanation for the emergence of energy homeostasis in bilaterians [21], cnidarians also possess muscular, nerve, and gastroderm or “stomach” cells, which contribute to the formation of an internal environment and rise of signaling factors important for homeostatic regulation, e.g. Mondo proteins and nuclear receptors.

### *MCR6* involvement in G6P recognition and transactivation

Initial models of Mondo and Mlx function were solely dependent upon the subcellular localization of these proteins. Since ChREBP, MondoA, and Mlx are largely cytoplasmic, it was predicted that nuclear transport would be sufficient for the transactivation of their gene targets. However, multiple experiments have shown that trapping ChREBP:Mlx or MondoA:Mlx in the nucleus, mutating the NES, or altering the phosphorylation of particular residues does not result in constitutive activation of reporter constructs [17], [19], [29].

Recently, MondoA nuclear accumulation has been attributed to both increased nuclear import, increased promoter occupancy, and decreased nuclear export in response to glucose derivative 2-DG [17]. ChREBP transactivational ability is also correlated to G6P abundance [31], suggesting that MondoA and ChREBP glucose response is directly mediated by G6P. Similarities in *MCR6* sequence with known G6P binding sites, and particularly the 6-phosphate molecule, strongly suggest that *MCR6* is an allosteric G6P binding region.

We defend that the putative function of *MCR6* in G6P allosteric activation and recruitment of coactivators is not mutually exclusive. Since MondoA and ChREBP have increased transactivation in response to G6P, its binding may trigger a conformational change that further exposes *MCR6* and facilitates cofactor interaction. The structure of GPI and Gfat1 proteins suggest that G6P binds within a largely hydrophilic pocket, while the 9aa TAD structure is variable and often disordered prior to forming an α-helix conformation upon cofactor binding [50]. The predicted structure of *MCR6* in MondoA and ChREBP displays an exposed pocket suitable for G6P binding as well as a flexible, coil region capable of making protein interactions (Figure S5).

### Model of G6P mediated Mondo Glucose Response

Based on our structure predictions and published sequence annotations, we propose the following model for Mondo glucose responsive transactivation. First, Mlx and Mondo family proteins readily form heterodimers within the cytoplasm, allowing Mlx:Mondo complexes to actively shuttle between the cytosol and nucleus. Second, *MCRV* interacts with the *LID* region, possibly through specific contacts with *MCRI*, *MCRIII*, and/or *MCRIV*, to block the transactivation region. Third, increased glucose and consequently G6P concentrations trigger signaling mechanisms that block the putative CRS in *MCRIV*. Fourth, G6P binding to *MCR6* causes an allosteric conformational change that “unlocks” *LID* and *MCRV* contacts, “pivots” *MCRII* so that it is buried, and “pins” *MCRI* in between the *LID* and *GRACE* so that Mondo remains in an open conformation. Finally, once in this open conformation, G6P may be released and cofactors such as CBP/p300 may bind to *MCR6* thereby activating Mondo proteins. In addition, non-vertebrate Mondo and ChREBP proteins interact with nuclear receptors, such as HNF4α, through the *NRB*, which activate these cofactors and increase transactivational potential. This model is in accordance with previous models based on protein manipulations as explained below.

First, MondoA and ChREBP monomers are confined to the cytosol and MondoA requires Mlx dimerization prior to nuclear localization [16], [17]. MondoA and ChREBP dimers have also been observed to actively shuttle between the nucleus and cytosol in numerous cell types (Table S1) and can be sequestered in the nucleus by NES inhibitor leptomycin B (LMB), whereas blocking MondoA and Mlx dimerization results in purely cytoplasmic monomers. Phosphorylation sites have been observed by mass spectrometry throughout ChREBP, except the *DCD/WMC* region, indicating Mlx dimerization is independent of phosphoregulation [27]. Conservation of *DCD/WMC* residues and similarity in both secondary and tertiary structure predictions implies monomer cytoplasmic retention and Mlx dimerization is consistent among Mondo family proteins. Thus it is likely that ChREBP and non-vertebrate Mondo proteins actively bind to available Mlx and are capable of shuttling to the nucleus as has been shown for MondoA.

Second, the *LID* region is responsible for regulating the otherwise constitutively active *GRACE* region in ChREBP. Inverting the *LID* and *GRACE* regions results in constitutive activation, showing the structural organization of these regions is important for ChREBP regulation [21]. Combinatorial deletions in ChREBP show *MCRII* has minimal repressive effects, while *MCRI*, *MCRIII* and *MCRIV* decrease transactivation in the presence of *MCRV* [22]. *MCRV* does not repress transactivation in the absence of *MCRI-IV*, yet mutations to *MCRV* increase transactivation when the *LID* is present [19]. Individual deletions of *MCRI-IV* were unable to alleviate low glucose repression [23], suggesting *MCRV* represses transcription conditionally upon multiple contacts within the *LID* region. From our structural prediction, it is likely the *MCRV* contacts *MCRIII* and *MCRIV* near residues S140 and S196, respectively (Fig. S5). These sites are known to affect the cytoplasmic localization of ChREBP as well as 14-3-3 binding, which is required for transactivation [19], [22]. Although MondoA and non-vertebrate Mondo proteins do not have these phosphorylation sites, our results based on sequence, domain, and structure similarity still support the notion that LID repression acts through multiple intramolecuar contacts and is common among all Mondo proteins.

Third, it has been suggested that MondoA *MCRIV* contains a CRS [18] and truncation mutants indicate it is located within the latter half of *MCRIV*. We find this region is highly conserved and likely to have the same interaction properties among Mondo proteins. Since increasing G6P abundance accelerates the rate of nuclear import for MondoA [17] and PP2A mediated dephosphorylation of S196 in ChREBP just downstream of *MCRIV* also results in increased nuclear abundance [25], we predict Mondo nuclear accumulation is, at least in part, goverened by a common mechanism, specifically G6P mediated relief of a CRS in *MCRIV*.

Fourth, it has been proposed that G6P allosterically affects the transactivation of MondoA and ChREBP [17], [23], [31]. *MCR6* provides an appropriate interface for G6P binding and also contacts the *LID* domain, particularly with *MCRIV* in our predicted structure. *MCRIV* is involved in general repression, where all mutants lacking this region show increased expression of reporters in a luciferase assay [21]. Additional deletion mutants show that *MCRI*, *MCRII*, and *MCRIII* are all necessary to overcome *MCRIV* repression and form an active complex. Thus G6P binding may break hydrogen bonds of *MCRIV* with these domains, thereby unlocking the repression of *GRACE* by *LID* and allowing these regions to separate.

Since glucose activated MondoA and ChREBP results in increased nuclear accumulation, we also expect the NES to be overpowered in high glucose medium. 14-3-3 binding has previously been attributed to blocking the NES, although *MCRII* is also necessary for recruiting a histone H3 acetyltransferase (HAT) cofactor. Since the *LID* region is not independently sufficient for MondoA or ChREBP transactivation [21], *MCRII* recruitment of a HAT cofactor must be a secondary effect. Based on the predicted N-terminus structure, it is plausible that *MCRII* pivots to make necessary contacts outside of the *LID* domain to help fix the separation between *LID* and *GRACE*.

*MCRI* is also required for glucose transactivation, but is not sufficient for full transactivation [22]. Hence *MCRI* may also form intrastructural contacts necessary for alleviating *LID* repression or interacting with activating cofactors. The position of *MCRI* near the interior of the predicted protein suggests it may act as a pin to wedge the *LID* and *GRACE* regions apart. Phosphorylation of S56 adjacent to *MCRI* increases ChREBP transactivational potential [27], possibly by facilitating this conformational change (Fig. S5).

*MCRIII* contains two essential regions. 14-3-3 and its binding region in *MCRIII* are required for ChREBP transactivation as is ChREBP:100–115 that is not necessary for 14-3-3 interaction. 14-3-3 has been shown to bind ChREBP constitutively [22], promote cytoplasmic retention, nuclear export, and transactivation. While the necessity of S140 phosphorylation for 14-3-3:ChREBP interaction is under contention [22], [29], it may affect the binding orientation as non-phosphorylated motifs can bind 14-3-3 in the opposite direction [53]. While S140 and S196 have been analyzed in ChREBP, we propose phosphorylation of the highly conserved T147/P148 site has a broader impact on Mondo family protein interactions and possibly affects 14-3-3 binding.

Moreover, the conserved *MCRIII* sequence corresponding to ChREBP:100–115 may affect Mondo phosphorylation status. According to the functional site prediction server ELM [54], this region matches a MAPK kinase-docking motif. Kinase docking domains are typically located 50–100 residues upstream of the phosphorylation site and characterized by a cluster of positively charged residues preceding a Φ×Φ hydrophobic sequence [55]–[57]. Conserved sequence 105-KWKxFKG**[LIV]**[KR]**L**-114 conforms to this motif, where positively charged residues are underlined and hydrophobic residues are in bold. Interestingly, W106 and F109 are invariant, and may contribute to interaction interface specificity. Moreover, 103-[ST]P-104 (human ChREBP numbering) residues directly precedes this motif in all Mondo sequences, but has not been identified as a phosphorylation site. Recent evidence also suggests that ChREBP activity in high glucose is dependent upon *O*-linked glycosylation, which targets sequences similar to phosphorylation motifs [58]. We anticipate the conditional status (e.g. phosphorylation, glycosylation, orientation, or intramolecular contacts) of these sites and domains are important for the activation of Mondo in response to changes in glucose levels.

Finally, MondoA and ChREBP recruit cofactors to promote transcriptional activation. Since mutants lacking the N-terminus have exceptionally high transactivational ability, G6P may only be necessary for relieving *LID* repression from *GRACE*. Hence G6P may be released from *MCR6* in the active/open conformation, thereby permitting *MCR6* access to cofactors. MondoA was shown to recruit a histone H3 acetyltransferase [17], while ChREBP is known to interact with CBP/p300 [39], which has histone acetyltransferase (HAT) function. *MCR6* matches the 9aa TAD motif depicting the CBP/p300 interaction region. Since *MCR6* is within the *GRACE* region, which is sufficient for transactivation [21], and mutating *MCRV* increases the transactivation potential [19], we deduce that *MCR6* acts as a TAD for Mondo proteins.

ChREBP and non-vertebrate Mondo transactivation may additionally rely on the interaction with nuclear receptors. Interestingly, nuclear receptors are specific to metazoans, and not found in sponges although present in cnidarians [59]. This agrees with our identification of Mondo proteins and the *NRB* motif.

Excluding MondoA, an LxQLLT sequence matching the *NRB* motif was conserved within the central region among non-vertebrate Mondo and ChREBP proteins. Tellingly, ChREBP, HNF4α, and CBP/p300 form a complex necessary for full activation of lipogenic enzyme L-PK. The HNF4α and ChREBP binding domains are directly adjacent within the promoter of this gene, indicating they are also juxtaposed within the complex. Since most nuclear receptors depend upon interaction with a *NRB* for activation, ChREBP may be fulfilling this role. This interaction may also help explain the relationship of activation between ChREBP and other nuclear receptors such as FXR and COUPTF-II [60].

In conclusion, MondoA and ChREBP are important glucose responsive genes involved in energy homeostasis. While ChREBP has evolved to have unique phosphoacceptor sites, the conservation of *MCRI-V*, *MCR6*, *bHLHZ*, and *DCD/WMC* domains indicates all Mondo family proteins are regulated by common mechanisms. Although their formal structure is not known, we predict their regulation is largely governed by intramolecular contacts. We further postulate that binding of G6P causes an allosteric conformational change, which forms an open, active complex where the *LID* repression is released from *GRACE* and permits interaction with coactivators such as CBP/p300.

## Methods

Full-length Mondo family protein sequences were obtained by surveying multiple genome databases as described in [12]. ClustalW, Dialign, and MAFFT were used to align the sequences and merged according to consensus regions and manual adjustment to construct a single, optimal alignment. Mondo Conserved Regions were specified as in [18] and depicted by weblogos [61].

### Sequence Conservation

Both the Jenson-Shannon Divergence (JS) score and entropy values were used to determine sequence conservation. For a multiple sequence alignment, the JS heuristic employs window-based extension that considers the conservation of sequentially neighboring sites and quantifies each score based on a weighted distribution of amino acids [34]. Hence the mutual information based JS score rates the conservation of each site by incorporating the autocorrelation of adjacent sites, where highly conserved sites have JS scores close to one and variable positions close to zero.

Entropy values were computed by the FastaEntropy program written by Andrew Fernandez. Entropy is a statistical measure of the amount of information or variation and, when applied to sequence alignments, can depict the conservation of sites, with lower entropy values signifying increased conservation [62]. Traditionally protein entropy is calculated by the Shannon Entropy equation based on the proportion of the 20 possible amino acids at each site. However, this method does not account for shared physicochemical properties among amino acids. To account for this, we also used a functional group entropy measure developed by [63] that is based on eight distinct categories of amino acids grouped according to physicochemical similarities. This method accentuates sites that are functionally constrained yet variable, e.g. conservation of I, V, L, M hydrophobic residues.

Site conservation is also highly correlated with structural and functional importance. To estimate and project the contribution of conserved sites on protein structures, we used the Consurf program available at http://consurf.tau.ac.il/ [64]. Consurf predicts functionally important regions in a given protein structure by estimating the phylogenetic relationship of homologs with similar known tertiary structure and ranking the evolutionary rate at each site [47]. Within this scheme, nine indicates site conservation and zero site variability.

### Identification of Functional Domains and Motifs

The presence of functional domains or motifs was determined by individually analyzing each sequence using multiple online tools. The presence of proline rich and glutamine rich regions was predicted by the Expasy program ScanProsite [65]. Additional motifs, such as the MAPK kinase docking domain, were predicted using regular expression patterns by the Eukaryotic Linear Motif resource (ELM) [54], while the 9aa TAD server was used to specifically evaluate putative CBP/p300 binding regions [50]. The MAPK docking motif in ELM is characterized by the regular expression [KR]{0,2}[KR].{0,2}[KR].{2,4}[ILVM].[ILVF], while the 9aa TAD regular expression is [GSTDENQWYM]{KRHCGP}[FLIVMW]{KRHCGP}{CGP}{KRHCGP}[FLIVMW][FLIVAMW]{KRHCP}; residues within brackets ‘[]’ are permitted and residues within braces ‘{}’ are prohibited.

### Characterizing the G6P recognition pocket

The structure of several G6P binding proteins has been crystallized, with specific attention to the G6P binding region, and desposited in the Protein Data Bank (PDB). During glucose metabolism in mammals, glucokinase (GK) or hexokinase (HKI-III) converts glucose to G6P [66]–[68], which can be reversed by G6P phosphatase (G6Pase) in the liver. G6P can be further metabolized by phosphoglucose mutase (PGM) to promote glycogen storage [69], [70], glucose phosphate isomerase (GPI) to produce fructose-6-phosphate (F6P) and continue in the glycolytic pathway [71], or G6P dehydrogenase (G6PDH) to enter the pentose shunt of glycolysis [72], [73]. Another enzyme, glutamine:fructose-6-phosphate amidotransferase (human: Gfat1, *E.coli*: Glms), can interact with G6P and F6P to promote the production of glycolipids through the glucosamine pathway [74]–[76].

We compared the G6P interacting residues described in the literature for each of these proteins to identify common features for metabolite recognition.

### Structural prediction of the *DCD* and N-terminal region of Mondo

Correctly predicting protein structures from amino acid sequences has been a goal within computational biology for the last several decades. The reliability of structure predictions often depends on the availability of homologous structure templates that allow for protein threading or homology modeling methods. These methods use a database of known structures to select a template with local or global similarities in secondary structure that can be used to fit the query model.

Secondary structure predictions for human, mouse, *C. elegans* and *Drosophila* Mondo sequences were formed by NPS@, which builds a consensus based on the individual secondary structure predictions of DPM, DSC, GOR1, GOR3, HNNC, MLRC, PHD, Predator, and SOPM programs [77]. Sequences exhibited similar secondary structure predictions with compatible alignments of alpha helices and beta sheets. We depict the secondary structure by the representative human ChREBP graphic (Figure 2) produced using Polyview [78].

While using structure prediction programs is straightforward, each method can form diverse structures and evaluating their accuracy is difficult. The metaserver 3D-jury addresses this concern by aggregating and comparing multiple structure predictions from several servers and ranking them based on structural similarity to create a more accurate consensus prediction [79]. Rosetta has also been accepted as a leading protein prediction software with particular application to *ab initio* design [80]. A structure prediction for ChREBP *DCD/WMC* was previously determined by The Human Proteome Folding Project using Rosetta and deposited at the yeast resource center [81], [82].

For determining the N-terminal structure, we used 3D-Jury on MondoA sequence 1–490 and ChREBP sequence 1–360. The 3D-Jury metaserver compares and ranks structural predictions from sequence only (EsyPred3, FFAS03, GRDB, Pfam-basic, Pfram-metabasic) and threading methods (3D-PSSM, FUGUE, INUB, mGenThreader, SAM-T02, samt06), whereby structure predictions are evaluated by the fit of each model and ranked according to their similarity to other models [79]. MondoA most closely matched the PDB structure (1p49A) of human estrone sulfatase using the INUB Hybrid Fold Recognition method with a Jscore of 29.67. The N-terminal protein structures were modeled by the program Modeller 9.1 [83] and images were produced by Chimera [84].

## Supporting Information

Figure S1 **ChREBP and non-vertebrate Mondo Secondary Structure.** Consensus secondary structure predictions are overlayed each sequence, with *MCRs* colored red and the *bHLHZ* and *DCD* domains colored blue. **A**) *H. sapiens* MondoA. The *PRR* is colored green **B**) *D. melanogaster* Mondo. The *NRB* is colored green.
(TIF)

Figure S2 ***DCD/WMC*** **entropy.** *DCD/WMC* region of all Mondo and Mlx sequences. Numbering corresponds to position in the alignment, shown in Figure 6. Low entropy values indicate site conservation for either a particular amino acid (red: AA) or physiochemical trait (black: FG), e.g. hydrophobic, although low entropy may also result from gaps in the alignment. The dotted line marks an arbitrary threshold of H = 0.1 to indicate highly conserved sites.
(TIF)

Figure S3 **DCD/WMC structure.** Rosetta and Human Proteome Folding Project prediction for ChREBP *DCD/WMC* domain. **A**) A cluster of five alpha helices is predicted within the *DCD/WMC* region of ChREBP. **B**) Hydrophobic (red) residues line the interior groove of α2, α3 and α4, while hydrophilic (blue) residues coat the exterior. **C**): Filled *DCD* structure in the same (left) and reversed (right) orientation as above, using Consurf conservation coloring (maroon: highly conserved, white: neutral, teal: variable). Highly conserved residues are labeled according to the human ChREBP sequence and the *WMC/DCD* alignment numbering.
(TIF)

Figure S4 **Sx[ST]xx[ST] motif locations.** We provide evidence that G6P may bind an Sx[ST]xx[ST] motif in Mondo proteins. This motif has low complexity and is found throughout Mondo sequences, but is only consistently conserved among species for MondoA (red), ChREBP (blue) and non-vertebrate Mondo (black) in the glucose-responsive region containing *MCR6*. Numbering corresponds to position in the alignment.
(TIF)

Figure S5 **ChREBP open and closed protein conformation.** Predicted structure of ChREBP in the closed (**A–C**) and open (**D–F**) conformation. Images **B** and **E** are 180 degree rotations of **A** and **D**, respectively, while **C** and **F** depict the structure from an overhead view. Domains are colored as for MondoA in Figures 8 and 9: *MCRI*-red, *MCRII*-orange, *MCRIII*-yellow, *MCRIV*-green, *MCRV*-purple, *MCR6*-blue. In addition, we have highlighted the proposed NES1 (light pink), the serine and threonine residues in *MCR6* (pale green), and the relevant and putative phosphorylation sites (magenta). Phosphorylation site S140 is located within *MCRIII* (A, D, C, F), S196 is downstream of *MCRIV* (C,F), while the putative phosphorylation sites S103 (near *MCRII*) and T147 are only accessible in the open conformation (D).
(TIF)

Table S1 **Cell type specific nuclear accumulation of MondoA and ChREBP in response to glucose.** Values represent the (∼approximate) percentage of cells with Mondo transcripts located in the cytoplasm (C), nucleus (N), or both (B) for low and high glucose medium in rat hepatocytes, 832/13 insulinoma cells, INS-1 pancreatic cells, L6 myoblasts, COS-7 and HEK293 kidney cells, and NIH3T3 fibroblasts.
(DOCX)
