# Supplementary figures and images for: A Novel N-Terminal Domain May Dictate the Glucose Response of Mondo Proteins
Source: PLoS One. 2012 Apr 10;7(4):e34803. doi: 10.1371/journal.pone.0034803 (PMC3323566; doi:10.1371/journal.pone.0034803)

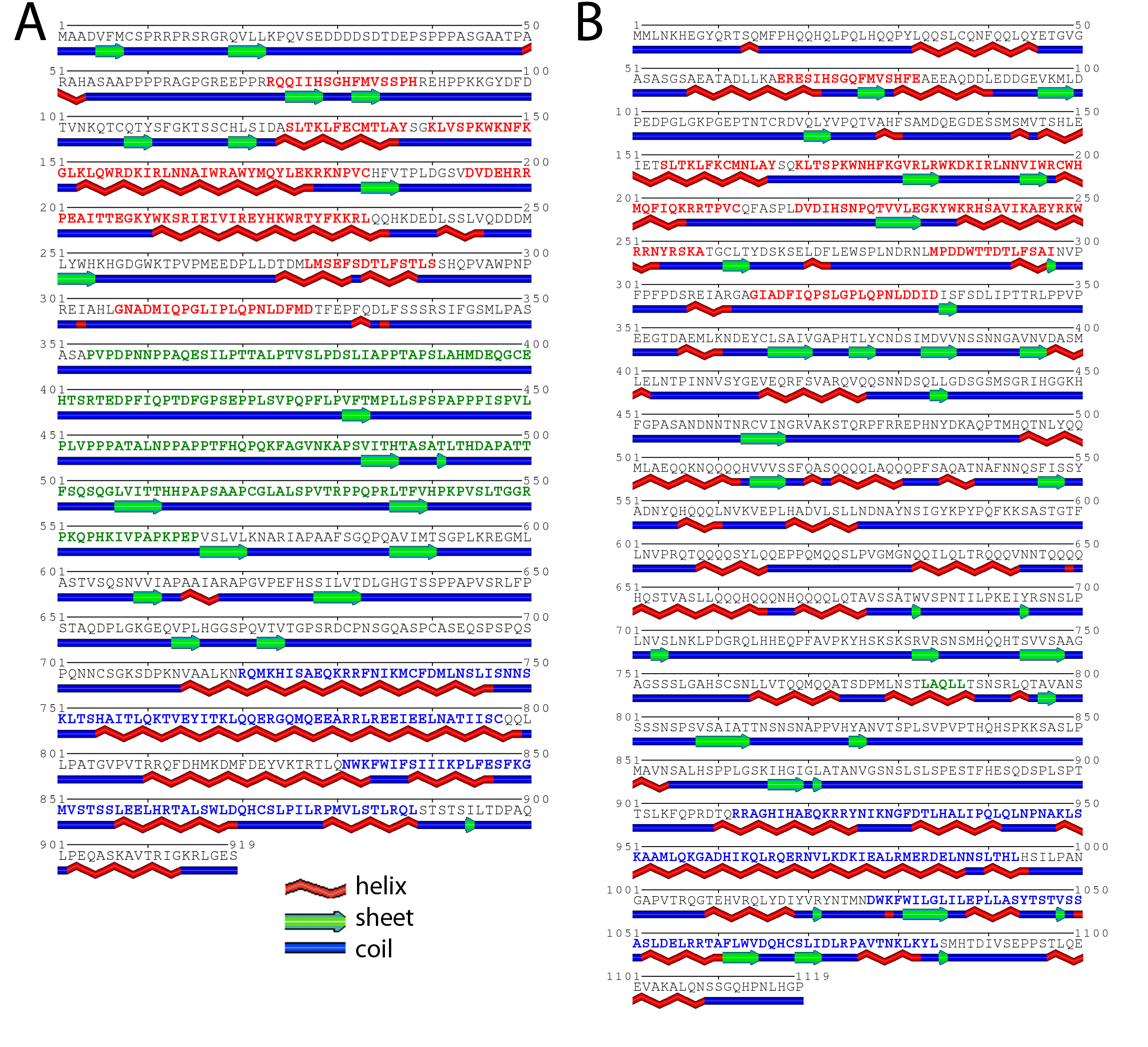

Supplement: Figure S1 — ChREBP and non-vertebrate Mondo Secondary Structure. Consensus secondary structure predictions are overlayed each sequence, with MCRs colored red and the bHLHZ and DCD domains colored blue. A) H. sapiens MondoA. The PRR is colored green B) D. melanogaster Mondo. The NRB is colored green. (TIF) [file pone.0034803.s001.tif]

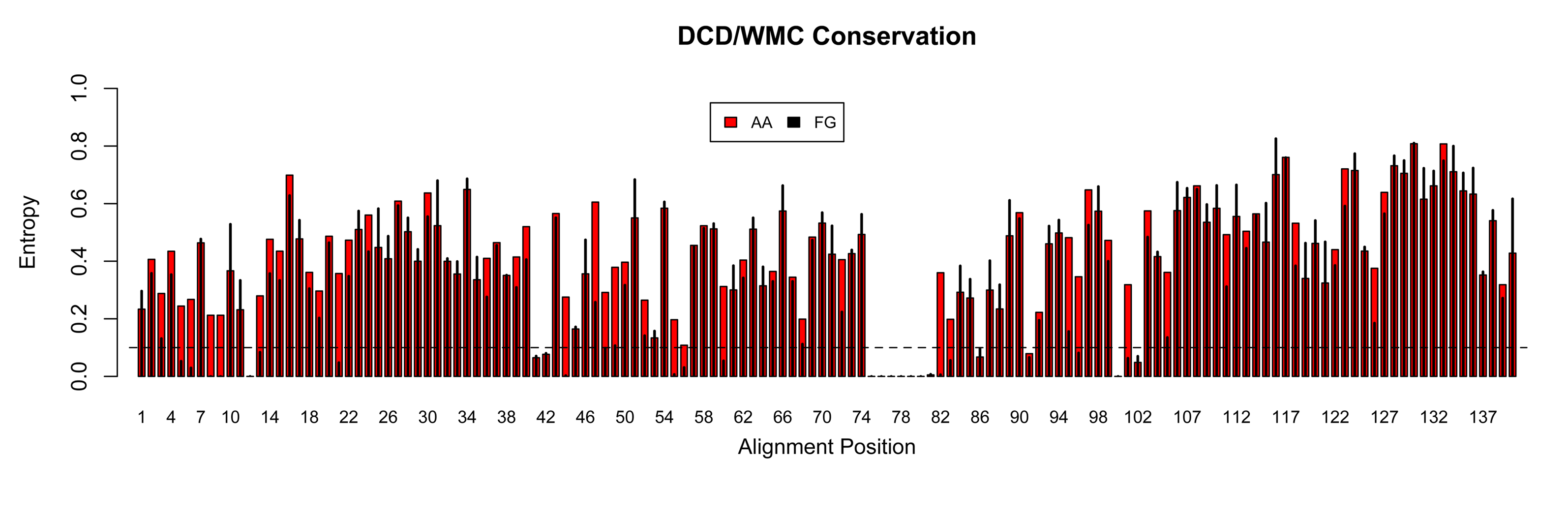

Supplement: Figure S2 — DCD/WMC entropy. DCD/WMC region of all Mondo and Mlx sequences. Numbering corresponds to position in the alignment, shown in Figure 6. Low entropy values indicate site conservation for either a particular amino acid (red: AA) or physiochemical trait (black: FG), e.g. hydrophobic, although low entropy may also result from gaps in the alignment. The dotted line marks an arbitrary threshold of H = 0.1 to indicate highly conserved sites. (TIF) [file pone.0034803.s002.tif]

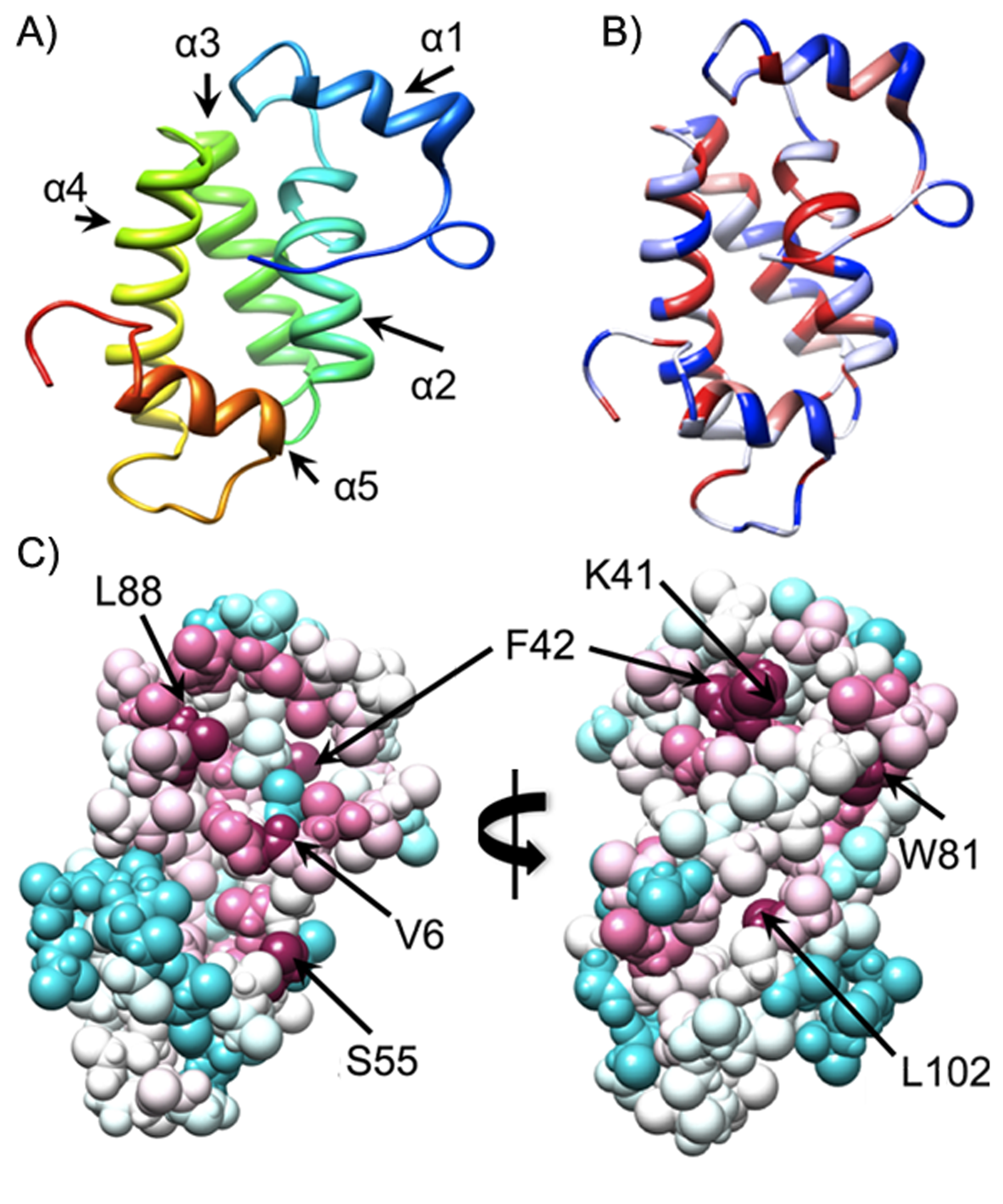

Supplement: Figure S3 — DCD/WMC structure. Rosetta and Human Proteome Folding Project prediction for ChREBP DCD/WMC domain. A) A cluster of five alpha helices is predicted within the DCD/WMC region of ChREBP. B) Hydrophobic (red) residues line the interior groove of α2, α3 and α4, while hydrophilic (blue) residues coat the exterior. C): Filled DCD structure in the same (left) and reversed (right) orientation as above, using Consurf conservation coloring (maroon: highly conserved, white: neutral, teal: variable). Highly conserved residues are labeled according to the human ChREBP sequence and the WMC/DCD alignment numbering. (TIF) [file pone.0034803.s003.tif]

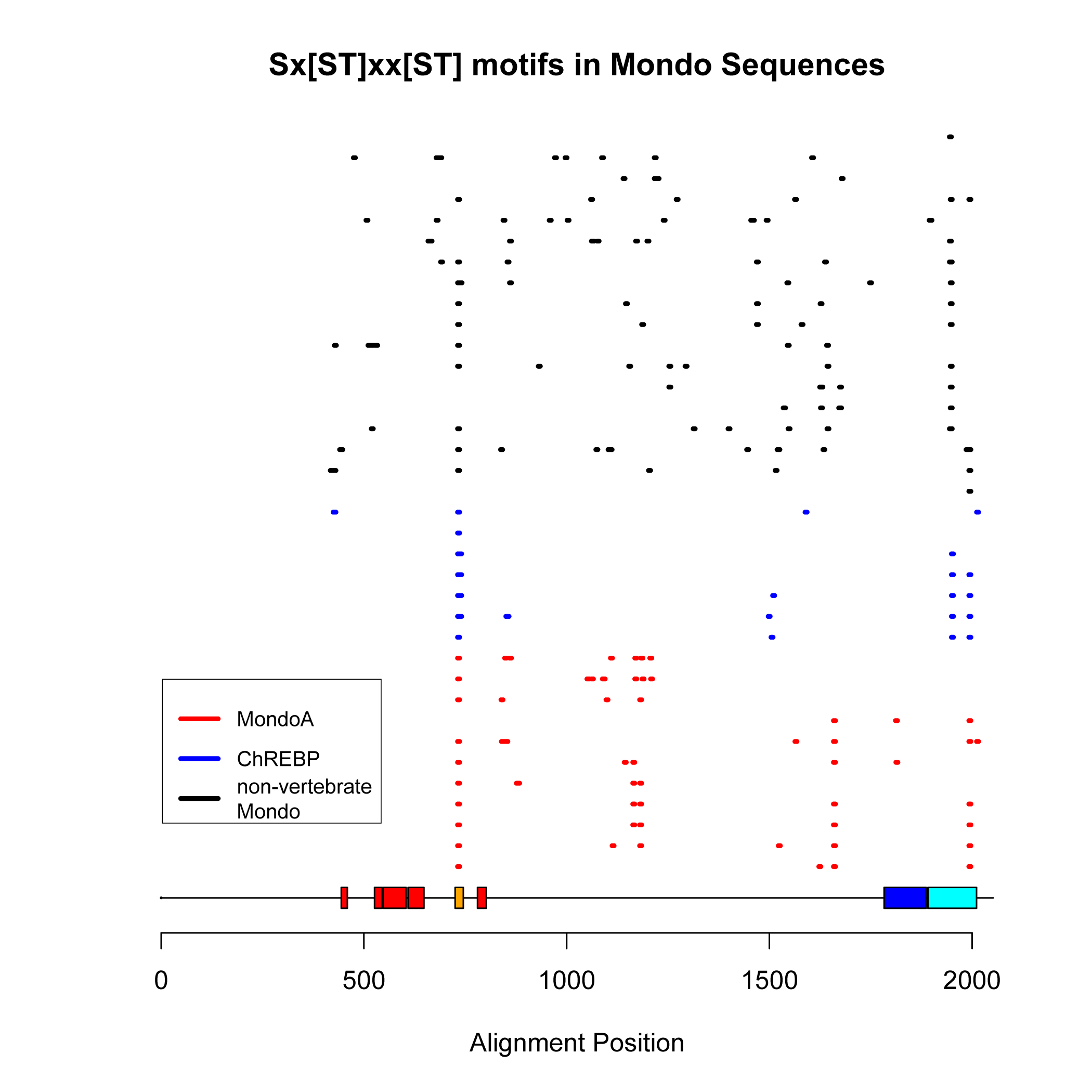

Supplement: Figure S4 — Sx[ST]xx[ST] motif locations. We provide evidence that G6P may bind an Sx[ST]xx[ST] motif in Mondo proteins. This motif has low complexity and is found throughout Mondo sequences, but is only consistently conserved among species for MondoA (red), ChREBP (blue) and non-vertebrate Mondo (black) in the glucose-responsive region containing MCR6. Numbering corresponds to position in the alignment. (TIF) [file pone.0034803.s004.tif]

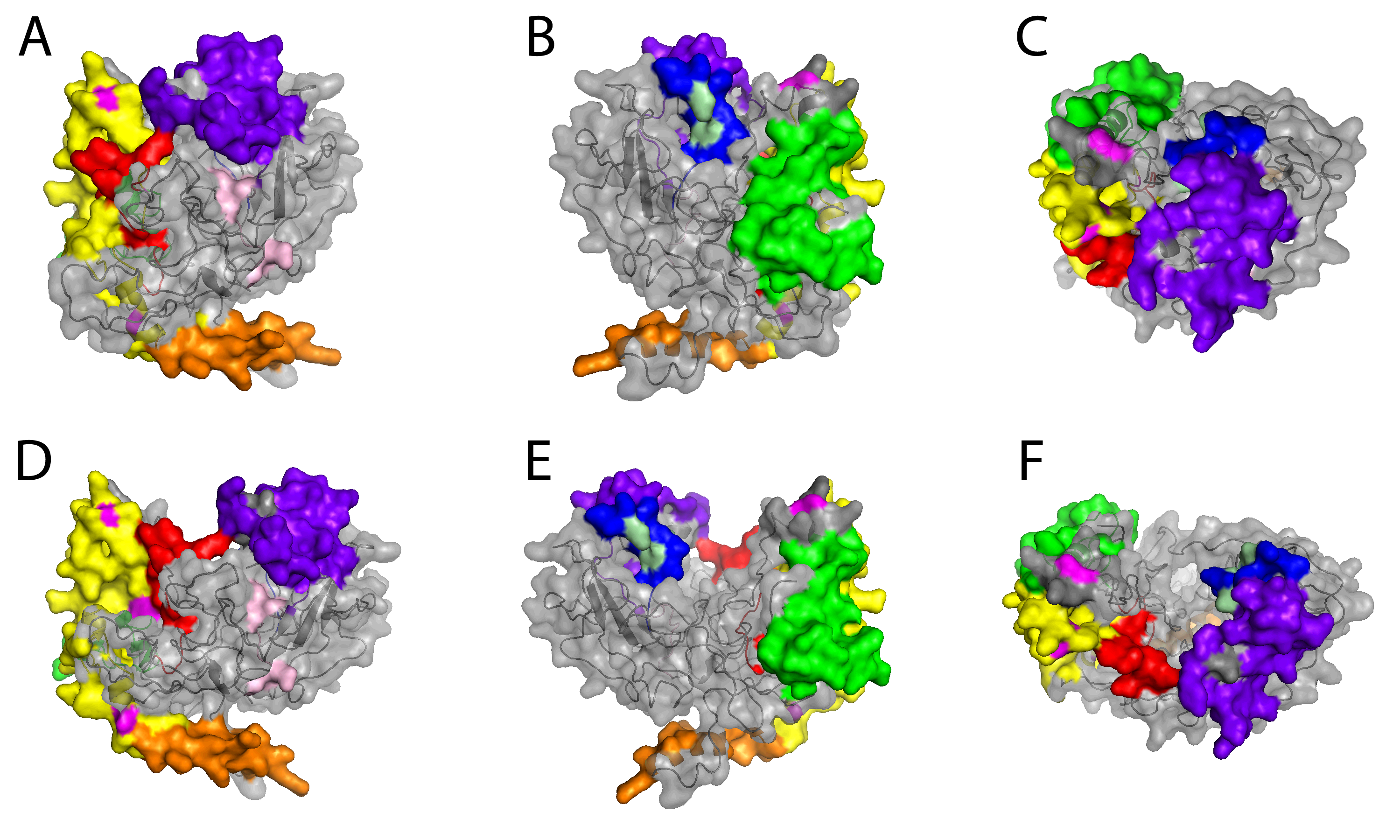

Supplement: Figure S5 — ChREBP open and closed protein conformation. Predicted structure of ChREBP in the closed (A–C) and open (D–F) conformation. Images B and E are 180 degree rotations of A and D, respectively, while C and F depict the structure from an overhead view. Domains are colored as for MondoA in Figures 8 and 9: MCRI-red, MCRII-orange, MCRIII-yellow, MCRIV-green, MCRV-purple, MCR6-blue. In addition, we have highlighted the proposed NES1 (light pink), the serine and threonine residues in MCR6 (pale green), and the relevant and putative phosphorylation sites (magenta). Phosphorylation site S140 is located within MCRIII (A, D, C, F), S196 is downstream of MCRIV (C,F), while the putative phosphorylation sites S103 (near MCRII) and T147 are only accessible in the open conformation (D). (TIF) [file pone.0034803.s005.tif]
