# Supplementary material for: A Novel N-Terminal Domain May Dictate the Glucose Response of Mondo Proteins
Source: PLoS One. 2012 Apr 10;7(4):e34803. doi: 10.1371/journal.pone.0034803 (PMC3323566; doi:10.1371/journal.pone.0034803)
Supplement: Table S1 — Cell type specific nuclear accumulation of MondoA and ChREBP in response to glucose. Values represent the (∼approximate) percentage of cells with Mondo transcripts located in the cytoplasm (C), nucleus (N), or both (B) for low and high glucose medium in rat hepatocytes, 832/13 insulinoma cells, INS-1 pancreatic cells, L6 myoblasts, COS-7 and HEK293 kidney cells, and NIH3T3 fibroblasts. (DOCX) [file pone.0034803.s006.docx]

**Supplemental Material**

**Table S1: Cell type specific nuclear accumulation of MondoA and ChREBP in response to glucose**

|  | **Cell line** | **Low Glucose (~5.5mM)** | | | **High Glucose (~27.5mM)** | | | **Reference** |
| --- | --- | --- | --- | --- | --- | --- | --- | --- |
| ChREBP |  | **C** | **B** | **N** | **C** | **B** | **N** |  |
|  | INS-1 |  |  |  | 72 | 23 | 5 | [27] |
|  | 832/13 | 94 | 6 | 0 | 78 | 18 | 2 | [19] |
|  | Rat hepatocyte |  |  | ~20 |  |  | ~45 | [29] |
|  | Rat hepatocyte |  |  | ~40 |  |  | ~80 | [25] |
|  | HEK293 |  |  | ~18 |  |  | ~48 | [28] |
| MondoA | L6 | ~95 | ~5 | 0 | ~10 | ~25 | ~65 | [17] |
|  | A549 | ~95 | ~5 | 0 | ~21 | ~24 | ~55 | [86] |
|  | HA1ER | ~95 |  | ~5 | ~15 |  | ~85 | [20] |

Values represent the (~approximate) percentage of cells with Mondo transcripts located in either the cytoplasm (C), nucleus (N), or both (B) for low and high glucose medium in the studies referenced.

*Mondo proteins have cell type specific nuclear accumulation*

Since MondoA and ChREBP are not currently known to have cytoplasmic activity, nuclear localization of these transcription factors is important for their function. Numerous studies have assessed their subcellular localization patterns in several cell lines, including glucose responsive rat hepatocytes [29, 33], 832/13 insulinoma cells, INS-1 pancreatic cells [23, 31], and L6 myoblasts [22], as well as COS-7 [26] and HEK293 kidney cells [32] and NIH3T3 fibroblasts [11, 16] that are not glucose responsive. Changes within these cellular environments are likely to affect MondoA and ChREBP glucose dependent functions including their subcellular localization and transactivation capabilities. For example, expression of ChREBP in rat hepatocytes localizes to the cytoplasm in low glucose conditions yet is mainly nuclear in high glucose [25, 29]. Similarly MondoA is predominantly cytoplasmic in low glucose, yet accumulates in the nucleus in high glucose in myoblasts and epithelial cells [17, 20, 86]. However, the expression of ChREBP remains highly cytoplasmic in both low and high glucose conditions in INS-1 and 832/13 cell lines [19, 21, 27]. The absence and minimal amount of nuclear ChREBP in pancreatic cells under low and high glucose conditions, respectively, suggests an increased export or decreased import system of ChREBP compared to other cell lines. Nonetheless, MondoA and ChREBP cellular localization patterns are similar among these cell lines implicating a common mechanism of glucose-dependent regulation among Mondo proteins.
